# Genome‐wide discovery of tissue‐specific miRNAs in clusterbean (*Cyamopsis tetragonoloba*) indicates their association with galactomannan biosynthesis

**DOI:** 10.1111/pbi.12866

**Published:** 2018-03-11

**Authors:** Anshika Tyagi, Deepti Nigam, Amitha Mithra S. V., Amolkumar U. Solanke, Nagendra K. Singh, Tilak R. Sharma, Kishor Gaikwad

**Affiliations:** ^1^ ICAR‐National Research Centre on Plant Biotechnology New Delhi India; ^2^ Present address: National Agri‐Food Biotechnology Institute Mohali India

**Keywords:** clusterbean, miRNAs, RNA‐Seq, qRT‐PCR, RA‐RT‐PCR, galactomannan

## Abstract

Owing to the presence of 80% soluble dietary fibre, high protein content and high value gum, clusterbean (*Cyamopsis tetragonoloba*) has recently emerged as an economically important legume. The developing clusterbean seeds accumulate 90% galactomannans in the endosperm and, therefore, can be used as a model crop to understand galactomannan biosynthesis and its regulation. miRNAs are tiny master regulators of their corresponding target genes, resulting in variations in the amounts of their metabolic end products. To understand the role of these regulators in galactomannan biosynthesis regulation, small RNA libraries were prepared and sequenced from five tissues of clusterbean genotype RGC‐936, and miRanalyzer and DSAP programs were used to identify conserved miRNAs and novel small RNAs. A total of 187 known and 171 novel miRNAs were found to be differentially expressed, of which 10 miRNAs were validated. A complicated network topology and 35% sharing of the target mRNAs between known and novel miRNAs suggest random evolution of novel miRNAs. The gene ontology (GO) annotation of potential target genes revealed the genes coding for signalling and carbohydrate metabolism (50.10%), kinases and other enzymes (20.75%), transcription factors (10.20%), transporters (8.35%) and other targets (10.6%). Two novel unigenes were annotated as ManS (mannosyltransferase/mannan synthase) and UGE (UDP‐ D‐glucose 4‐epimerase) and validated as targets for three novel miRNAs, that is *Ct*‐miR3130, *Ct*‐miR3135 and *Ct*‐miR3157. Our findings reveal that these novel miRNAs could play an important role in the regulation of the galactomannan pathway in *C. tetragonoloba* and possibly other galactomannan‐producing species.

## Introduction

Clusterbean or guar (*Cyamopsis tetragonoloba*) is an economically important, drought‐tolerant annual legume crop belonging to the family Leguminosae, mostly grown in the semiarid regions of India. It is assumed to have evolved from the African species *Cyamopsis senegalensis* having chromosome number 2*n*=14 (Mudgil *et al*., 2014). It is a highly nutritious crop with 80% soluble dietary fibre on a dry weight basis (Shaikh and Kumar, 2011). The endosperm contains 90% gum, known as guar gum, used as a stabilizer in food, pharmaceutical and mining industries. Galactomannans/glucomannan are hemicellulosic polysaccharides composed of a (1→4)‐linked β‐D‐mannan backbone substituted with single‐unit (1→6)‐linked α‐D‐galactosyl residues (Mudgil *et al*., 2014). They are known to accumulate in large quantities in the seed endosperms of many leguminous plants (Reid, 1985) and seeds of some nonleguminous species, such as *Cocos nucifera, Elaeis guineensis, Coffea arabica, Phytelephas macrocarpa* and *Phoenix dactylifera*. A brief review of the leguminous and nonleguminous plants possessing galactomannan is given in Table S1.

Small RNA/microRNAs (miRNAs) are universal and highly conserved 20–24 nucleotides (nt) long noncoding RNAs that have been shown to play a vital role in growth, development and metabolic regulation of several organisms, including plants. To date, nine species of the Leguminosae family have been reported to contain varying numbers of mature and precursor miRNAs as given in Table S2 (Kozomara and Griffiths‐Jones, 2014). A diverse set of miRNAs and other types of small RNAs have been identified from the developing rice grains (Lan *et al*., 2012). The sequencing of sRNA populations from soybean seeds and vegetative tissues has also revealed tissue‐preferential expression of certain miRNAs (Zabala *et al*., 2012). [Correction added on 13 April 2018, after first online publication: Soya bean has been corrected to soybean in the preceding statement and throughout this current version.] Thus, the spatiotemporal regulation of tissue‐ and cell‐specific genes as well as the miRNAs may have played imperative roles in morphological and developmental variation during evolution in various organism including plants (Ha *et al*., 2008; Ng *et al*., 2011; Niwa and Slack, 2007; Graeber *et al*., 2013).

Deep‐sequencing technologies quickly reveal the hidden information of a genome. Prediction and characterization of miRNAs using *in silico* approaches are emerging as faster, and trustworthy methods as compared to laboratory‐based cloning (Gupta *et al*., 2017; Nigam *et al*., 2015). So far, genomewide discovery and characterization of miRNA have been reported for leguminous plants such as *G. max, M. truncatula* and *P. vulgaris* (Formey *et al*., 2015). However, despite its economic importance, very little work has been done on the characterization of the clusterbean at the genomic and transcriptomic levels. Recently, transcriptomic analysis for different tissue has been performed with the identification of more than three thousand SNPs and SSRs (Tanwar *et al*., 2017; Rawal *et al*., 2017). However, these workers did not identify miRNA from their transcriptomic data. Only a few studies have been conducted to determine genetic diversity in guar, and hence, the development of genomic resources in guar is limited (Ibrahim *et al*., 2012; Kumar *et al*., 2013; Kuravadi *et al*., 2014; Manivannan and Anandakumar, 2013; Manivannan *et al*., 2015; Morris, 2010; Pathak *et al*., 2011; Punia *et al*., 2009; Sharma *et al*., 2014a,b; Singla *et al*., 2016; Tanwar *et al*., 2017; Wadhwa *et al*., 2015).

The present work was undertaken to sequence five small RNA libraries from three vegetative and two reproductive tissues of the developing plants of clusterbean genotype RGC‐936 and identify known and novel miRNAs with the objective to examine their association with the galactomannan biosynthesis pathway at the target level.

## Results

### Overview of small RNA library sequencing and mapping

**Figure 1:**
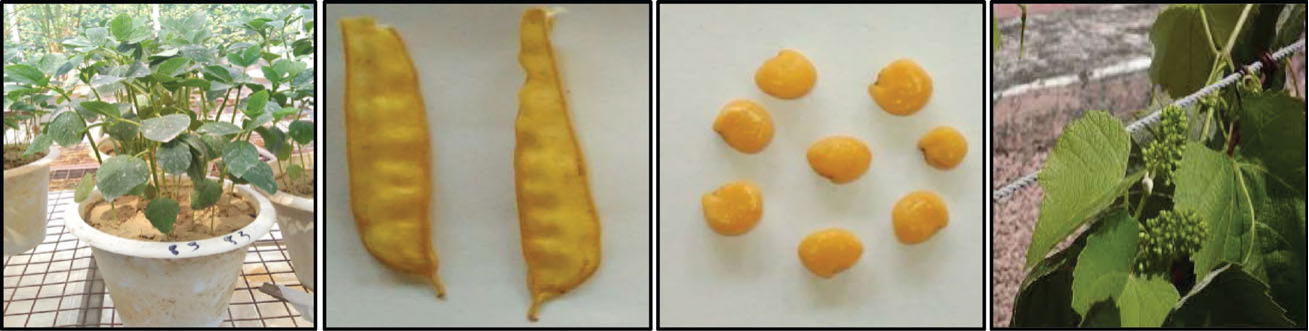
Clusterbean tissue samples used for miRNA isolation and preparation of the Illumina small RNA‐seq libraries grown in the net house, NRCPB, New Delhi, India.

**Figure 2:**
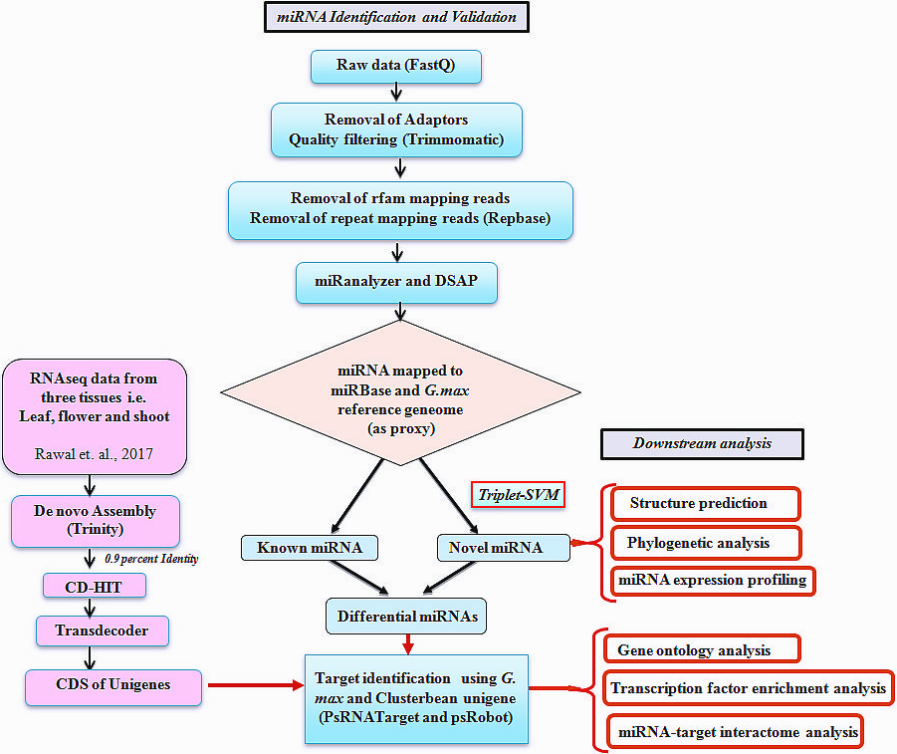
Systematic representation showing the workflow of the known novel miRNA and their target identification in the clusterbean using two softwares miRanalyzer and DSAP, and their downstream analysis. [Correction added on 13 April 2018, after first online publication: bud has been corrected to flower in this figure.]

**Figure 3:**
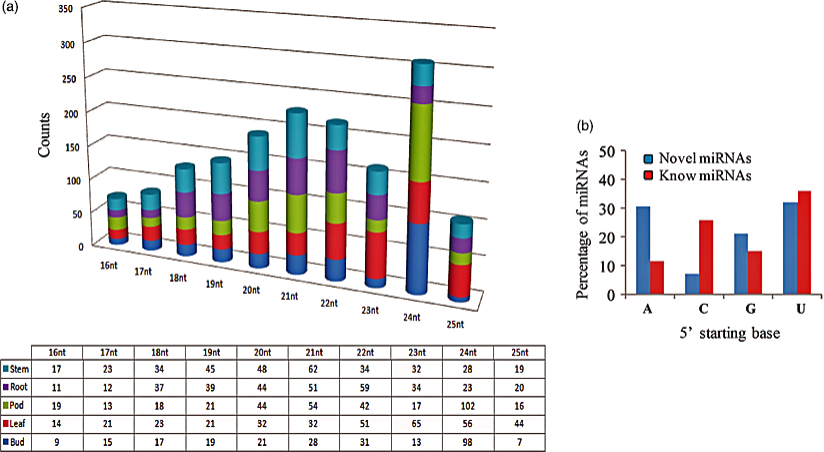
Characteristics of the known and novel clusterbean miRNAs. (a) Length distribution of the known and novel miRNAs (as pooled) in five different tissues. (b) Comparison of the 5′ nucleotide of the novel and known miRNAs.

Quality control (QC)‐passed RNA samples from root, stem, leaf, bud and seed tissues were used as starting material for sequencing of the clusterbean genotype, RGC‐936 miRNAs (Figure 1). Sequencing was carried out on an Illumina NextSeq 500 using 1 × 75 based chemistry. The means of the libraries fragment size distributions of clusterbean tissues for the root, stem, leaf, bud and seed were 149 bp, 158 bp, 154 bp, 148 bp and 156 bp, respectively. From the five libraries, approximately 116 million raw reads of a total size of 3.17 GB were obtained and further analysed. The raw data were generated as 21 431 269 (root), 23 976 233 (stem), 18 318 769 (leaf), 26 122 124 (bud) and 26 683 426 (seed) reads for each tissue, as given in Table 1. The raw sequence data have been submitted to NCBI (http://www.ncbi.nlm.nih.gov/) as Bioproject accession PRJNA390810. The workflow diagram of the analysis done is shown in Figure 2. Following removal of the low‐quality and adapter sequences, there were 12 351 758, 22 024 040, 13 834 891, 17 547 046 and 21 030 489 clean reads (total ~88 million) obtained from the root, stem, leaf, bud and seed, respectively (Table 1). The reads were quality‐filtered, and the adaptors were removed from the data. The ribosomal RNAs (rRNAs), transfer RNAs (tRNAs), small nuclear RNAs (snRNAs) and small nucleolar RNAs (snoRNAs) were also filtered out from the data (Table 1; Figure 1). To characterize the clusterbean small RNAs, the length distributions of the pooled miRNAs (known and novel) were analysed and plotted as shown in Figure 3a. Among these filtered reads, most of the redundant reads were found to be 21 nt in length, whereas the length distribution of the unique sequences showed that the most abundant sequences were 24 nt, accounting for approximately 39%. The dominance of the 24 nt read length is consistent with previous reports from other species, including *Arabidopsis thaliana*,* Oryza sativa, Solanum lycopersicum, Zea mays, Medicago truncatula*,* Cucumis sativus* and *Populus trichocarpa* (Fahlgren *et al*., 2007; Jiao *et al*., 2011; Martinez *et al*., 2011; Morin *et al*., 2008; Moxon *et al*., 2008; Puzey *et al*., 2012; Szittya *et al*., 2008). Moreover, the distribution of 5′ starting bases in known and novel miRNAs (pooled from all five tissues) was quite relational, wherein the known and novel miRNAs (miRBase database) had 38.22% and 32.10% with 5′U, 15.10% and 20.15% with 5′G, 10.01% and 30.00% with 5′A and 26.99% and 7.01% with 5′C, respectively (Figure 3b).

**Table 1:** Pre‐assembly statistics of clusterbean small RNA‐Seq data from five tissues

Tissues	Raw reads	Adaptor removal/Trimmed Reads	HQ reads	Reads dropped	Unique contigs/tags	Rfam filtered reads	Rfam unique contigs
Bud	26 122 124	17 910 938	17 547 046	363 892	1 617 067	7 468 403	233 299
Leaf	18 318 769	14 180 321	13 834 891	345 430	3 211 242	3 253 598	129 030
Seed	26 683 426	21 449 199	21 030 489	418 710	1 043 017	11 305 495	249 123
Root	21 431 269	12 650 284	12 351 758	298 526	2 339 937	6 070 385	367 936
Stem	23 976 233	22 430 228	22 024 040	406 188	4 024 523	8 602 117	279 801

### Identification of known, conserved and novel miRNAs

To predict miRNAs, all unique reads (gained after filtering) were submitted to the miRanalyzer and mapped (with no mismatch) against the reference genome of *G. max* using the Burrows–Wheeler transform (BWT)‐based algorithm, Bowtie with a mismatch <2 (Etebari and Asgari, 2014). For each library, 50%–68% of the reads mapped to the *G. max* genome (Table 2). To recover a putative miRNA candidate, the potential precursors were subjected to a series of stringent criteria suggested for the annotation of the plant miRNAs (Ambros *et al*., 2003; Kozomara and Griffiths‐Jones, 2014; Meyers *et al*., 2008). Finally, the sequences with no homology to any previously known and conserved plant miRNAs were denoted as novel miRNAs in clusterbean. We also obtained small RNA sequences that have been validated as potential precursor sequences from the triplet‐SVM classifier (Liu *et al*., 2015). Deep‐sequencing small RNA analysis pipeline (DSAP), a web‐based miRNA discovery pipeline, was additionally used for the identification of potential known miRNA candidates that perfectly matched with the mature miRNAs of soybean (miRBase 21) with a 100% BLAST sequence identity and full‐length parameters ‘‐ F F ‐W 16′ (to turn off the low complexity filter and increase the word size to 16 for increased speed). The sequences that matched with miRBase entries of other plant species were designated as conserved miRNAs. Using both the miRanalyzer and DSAP software programs successively, a total of 508 potential known miRNAs, representing 26 families and 293 novel miRNAs, were identified in the clusterbean. The count information of the known and novel miRNAs is given in Tables S3 and S4.

**Table 2:** Summary of the reference genome mapping of the clusterbean microRNA reads against *Glycine max*

Sample	Reads	Reads mapped	Per cent mapping
Bud	17 547 046	11 151 562	63.55236
Leaf	13 834 891	7 805 049	56.41569
Seed	21 030 489	10 593 180	50.37058
Root	12 351 758	8 416 637	68.14121
Stem	22 024 040	12 487 658	56.70012

### Abundance of conserved miRNA families in five different tissues of clusterbean

**Figure 4:**
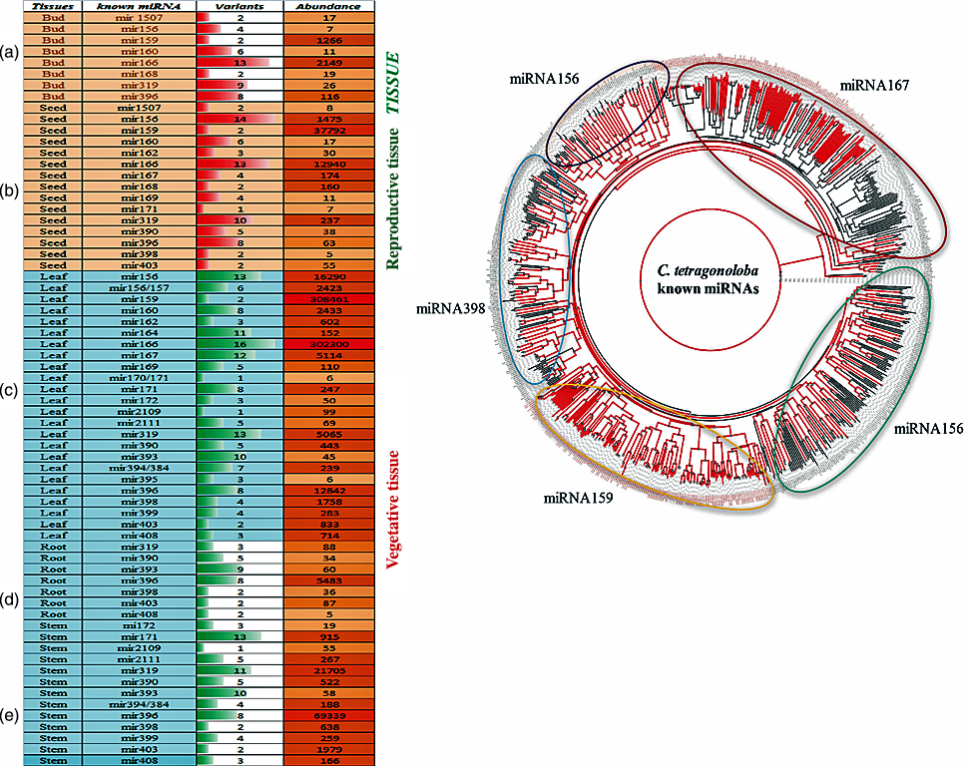
Quantitative and qualitative analysis of the known miRNA families (a) known miRNA variants and their abundance in each family in five different tissues, that is bud (a), seed (b), leaf (c), root (d) and stem (e); (b) A phylogenetic analysis of *C. tetragonoloba* known miRNAs (with red colour lines) showing strong conservation with its closest species, viz. *Medicago tranculata, Cajanus cajan and Glycine max* (black colour).

The abundance of miRNAs varied from 5 to 69 339 reads among miRNA families in different tissues (Table S3). The total number of sequence reads from all the miRNA variants (>5 reads) within each miRNA family was pooled for abundance analysis. Within each family, the proportion of total reads was accounted for by the major variants. The total number of reads for each family and the number of variants are shown in Figure 4a. In the seed tissue, of 15 miRNA families, miRNA159 was observed as the most abundant, followed by the miR166, miR156, miR319, miR167 and miR168, accounting for 71.3%, 10.9%, 2.8%, 0.4%, 0.3% and 0.3%, respectively. The remaining miRNA families together accounted for approximately 14% of the total conserved miRNA reads. The number of miRNA variants in each miRNA family of five different clusterbean tissues was highly variable. For example, there were 6, 43, 37, 46, 12 and 4 unique variant sequences (with reads ≥ 100) in the miR159, miR166, miR156, miR319, miR167 and miR168 families, respectively. Our data showed that miRNAs mir403, mir398, mir396, mir390, mir319, mir171, mir168, mir167, mir166, mir162, mir160, mir159, mir156 and mir1507 were highly expressed in at least one or more tissues. The miRNAs mir168 and mir1507 were found to be preferentially expressed in the reproductive tissues (seed and bud), while mir396 and mir319 were found to be involved in both the vegetative and the reproductive tissues (leaf, root, stem, seed and bud). The homology searches against miRNA precursor sequences of three legume species, namely *G. max*,* Cajanus cajan* and *M. truncatula,* identified some functionally important families such as miR156, miR319, miR166, miR159, miR398 and miR167 (Figure 4b).

### Expression dynamicity of miRNAs in different tissues

**Figure 5:**
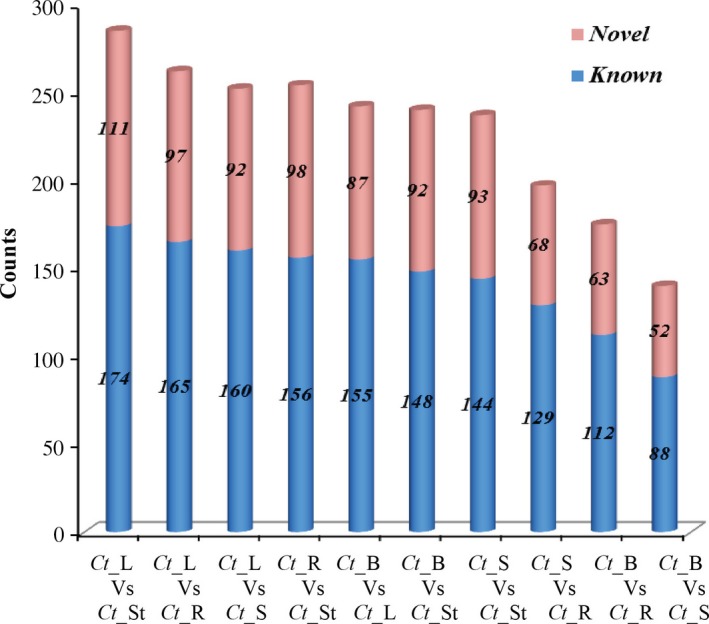
Number of differentially expressed known (blue colour) and novel miRNAs (pink colour) among the comparisons of five tissues.

**Figure 6:**
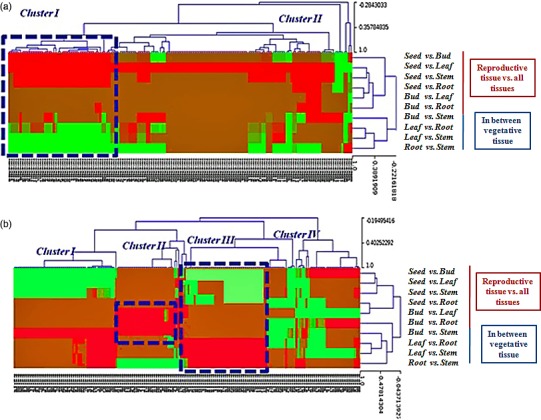
Heat map and unsupervised hierarchical clustering showing (a) 171 novel and (b) 187 known tissue‐specific and tissue‐enriched differentially expressed miRNAs of *C. tetragonoloba* in different tissues (samples).

Based on the sequencing data with log2‐ratios values, a maximum of 178 miRNAs (144 known and 34 novel) were observed as neutral in expression, 1320 (866 known and 454 novel) were up‐regulated (log2 > +1, *P *<* *0.05), and 786 (421 known and 365 novel) were down‐regulated (log2 < −1, *P *<* *0.05; Figure 5; Tables S11 and S12). Interestingly, 187 known and 171 novel miRNAs were found as unique (after removal of the duplicate reads) and differentially expressed. Using the uncentred Pearson correlation as the distance metric, an unsupervised hierarchical heatmap was obtained (Figure 6a, b). Two clusters were formed, that is Cluster I and Cluster II, for the novel miRNAs. Overall, the expression pattern of the seed contrasted with the three vegetative tissues, that is leaf, stem and root, as some miRNAs were unique, which is consistent as well as higher in expression (Figure 6a). Among these, a dense cluster of 25 quantitatively co‐expressed miRNAs was observed. After correlating the miRNA‐target ontological relationship, we found that this cluster mainly consists of miRNAs targeting the core galactomannan synthesis machinery, that is the genes coding for the enzymes needed to synthesize the mannan backbone (mannan synthase, ManS) and the genes that synthesize the galactosyl side chains (galactosyltransferase, GMGT), modulate the postdepositional degree of galactose substitution (α‐galactosidase) and produce the nucleotide sugar building blocks UDP‐galactose and GDP‐mannose (UDP‐glucose 4′‐epimerase and mannose‐1P guanylyl transferase), respectively.

The hierarchical clustering of the known miRNAs showed four patterns, and thus, four different clusters (clusters I, II, III and IV) were formed (Figure 6b). Only clusters I and III showed consistent miRNA expression. Cluster II showed higher as well as distinct/unique expression patterns of miR396, miR398, miR403, miR408, miR167, miR319, miR393 and miR396 in buds. Significant expression of these miRNAs was not found in seeds and other vegetative tissues. Cluster III contains some biologically important miRNA families such as miRNA156/157, miRNA160, miRNA164 and miRNA166 that show contrasting expression behaviour, that is higher expression in the leaf but lower expression in the seed and insignificant expression in the bud tissue.

### miRNAs–Transcription factors crosstalk

**Figure 7:**
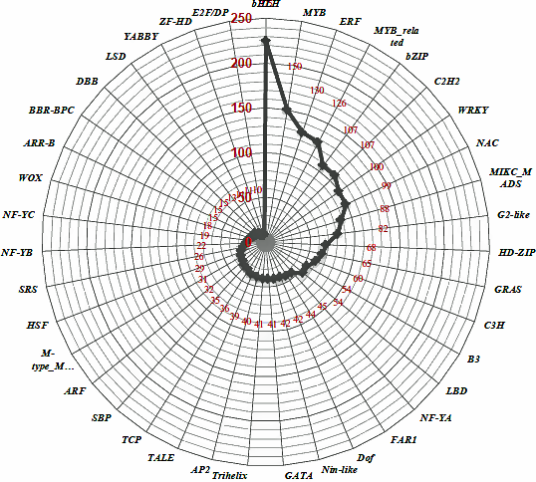
Enrichment of the 39 transcription factors families identified as miRNA targets in *C. tetragonoloba*.

**Figure 8:**
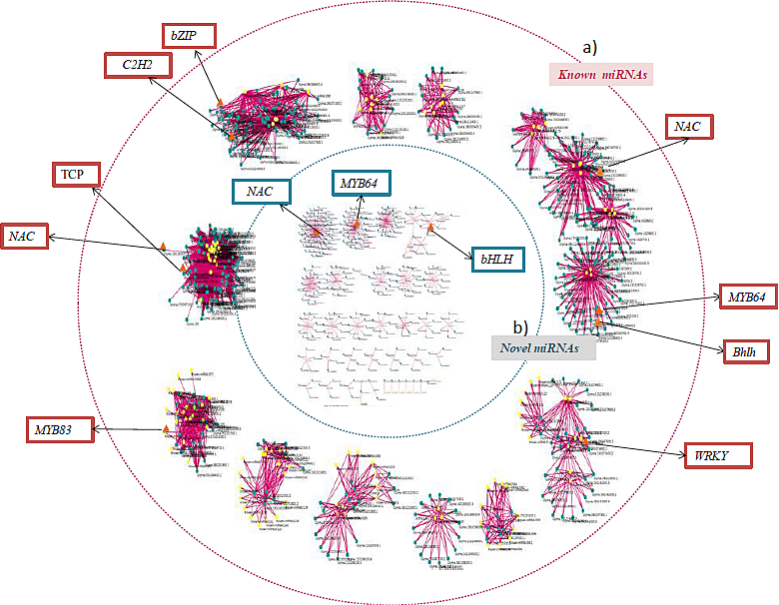
Topological differences observed within the (a) known and (b) novel miRNAs target communities of clusterbean. The miRNAs are shown in the yellow colour, while their targets are in the blue colour. The enriched TFs identified within each community of known and novel miRNAs are represented within red boxes (outside the circle) and blue colour boxes (inside of the circle), respectively.

Annotation of miRNA‐targeted unigenes revealed enrichment of total 39 TFs such as bHLH (count 250), Myb (count 150), Myb related (count 126), ERF (count 130), Bzip and C2H2 (count 107), WRKY (count 100) and NAC (count 99; Figure 7). Further, a strong connectivity was observed within a hub (made up of miRNAs and targets) and in‐between the hubs for three types of interactions: (i) 2 or >2 miRNAs, (ii) 1 miRNA with >1 targets and (iii) >1 miRNA >1 targets. A total of 11 such compactly associated miRNA‐target communities were observed for known miRNAs, whereas 16 communities (less compact) were identified for the novel miRNAs (Figure 8). Interestingly, 35% of the commonly regulated target genes were observed between the known and novel miRNAs. Each hub's community was governed by its respective TFs, that is NAC, MYB64, MYB83, bHLH, WRKY, TCP, C2H2 and bZIP (Figure 8). An overexpression of three common TFs, specifically NAC, MYB64 and bHLH, was observed between the novel and known miRNA hub communities.

### Ontological annotation of potential miRNA target

**Figure 9:**
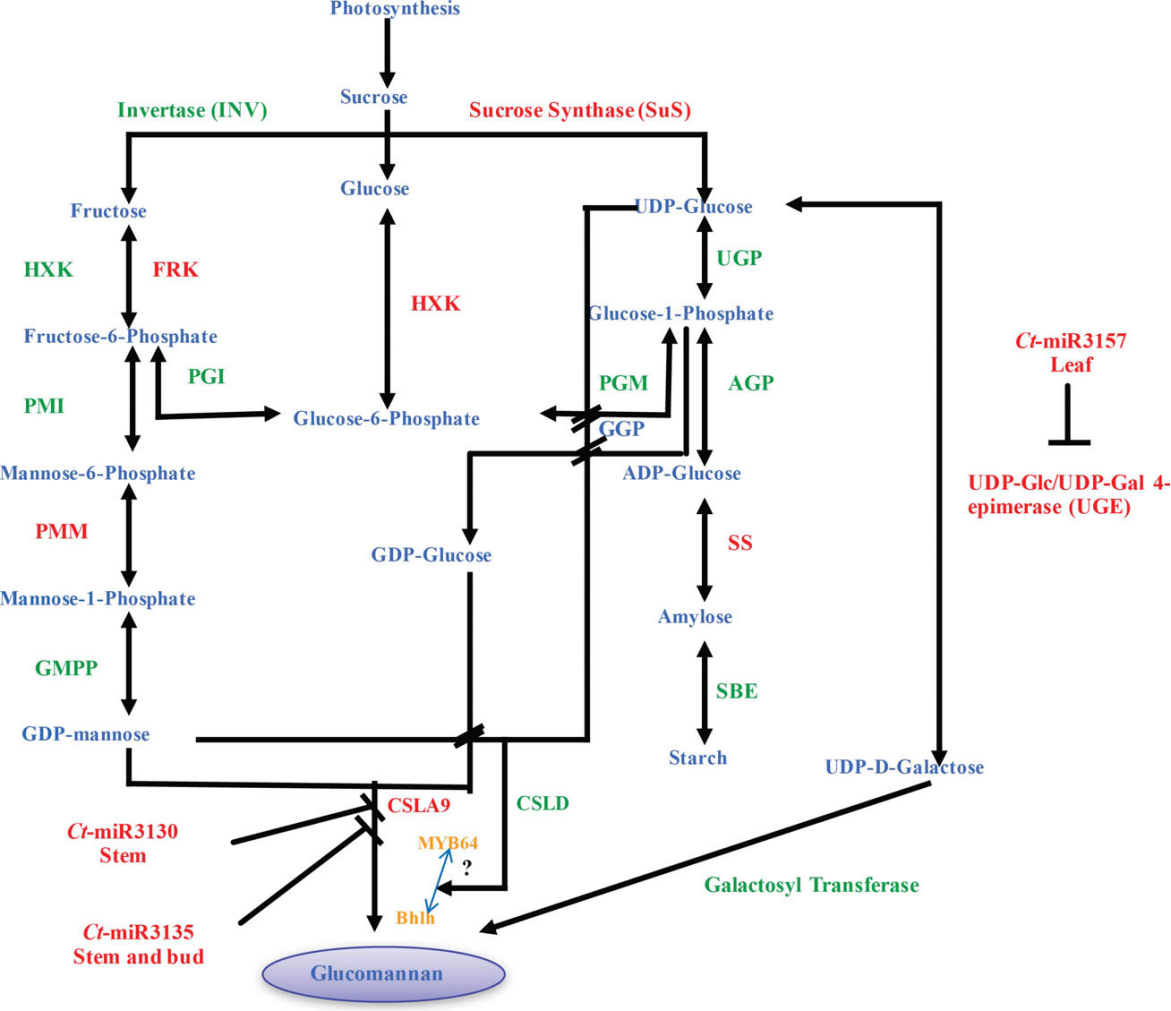
Schematic illustration of the galactomannan pathway involving enzymes in the galactomannan biosynthesis pathway. Identified enzymes within the unigenes data set are highlighted in red, while products are in blue. The miRNAs identified and validated via RT‐PCR are shown in pink. The two transcription factors, MYB64 and bHLH, found to regulate the CSLA9, that is mannan synthase 9, are shown in orange.

With the *G. max* as a reference target database, in total 169 known and 120 novel miRNAs, governing their 532 and 421 target genes, were acquired using psRNATarget, whereas 154 known and 159 novel miRNAs targeting 602 and 513 corresponding target genes, respectively, were obtained after processing with psRobot. Subsequently, 495 and 325 common targets were predicted for 172 known and 47 novel miRNAs (Tables S5 and S6). Interestingly, using the coding sequences from the clusterbean unigenes derived from the pooled data of leaf, flower and shoot tissues (Rawal *et al*., 2017; Figure 1), a total of 1892 and 832 targets for 177 known and 68 novel miRNAs, respectively, were obtained as two common sets with these two programs (Table S7). [Correction added on 13 April 2018, after first online publication: bud has been corrected to flower in the preceding statement.] SEA (singular enrichment analysis)‐based ontological enrichment analysis (Fisher's exact test, FDR‐corrected *P*‐value ≤ 0.05) of these common miRNA‐targeted genes showed their categorization into 280 biological processes, 68 cellular components and 453 molecular functions (Tian *et al*., 2017). A concordance gene annotation and categorization in conformity with the biological pathway (Biology Process), cellular localization (Cellular Component) and molecular activity (Molecular Function) were observed, analysed and reported in Tables S8, S9 and S10. A large spectrum of miRNA‐regulated biological processes was observed that majorly consists of signalling and carbohydrate‐related genes (50.10%), kinases and other enzymes (20.75%), transcription factors (10.20%), transporters (8.35%) and others (10.6%; Figure S1). Interestingly, a higher count for the enzyme classes (directly or indirectly related to galactomannan biosynthesis pathway) was found to be regulated *via* these known and novel miRNAs (Table 3). Hence, these previously unexplored clusterbean miRNA‐target unigenes were annotated and classified as enzyme families that were majorly involved in governing the galactomannan biosynthesis pathway (Table S13, Figure 9). Furthermore, only 156 miRNAs (98 known and 58 novel) and 765 targets were commonly assessed between *G. max* and clusterbean, suggesting the applicability of our unigene data.

**Table 3:** Identification of miRNA‐targeting enzymes using clusterbean unigenes

Enzymes (Unigenes)	Unigene Counts	Known/Novel miRNAs
Sucrose synthase	38	Ct‐miR482
Starch synthase	2	*Ct*‐miR159
UDP‐Glc/UDP‐Gal 4‐ epimerase(UGE)	38	*Ct*‐miR3157 (novel)
Glycosyl transferase	327	*Ct*‐miR160 (Known)
Callose Synthase (CALS)	34	
Cellulose synthase	175	*Ct*‐miR3130 (known), *Ct*‐miR3135 (Novel)
Mannosyltransferase	54	–
Mannosidase	67	*Ct*‐miR482
Galactosyl transferase	42	–
Phosphomannose isomerase	7	–
GDP‐mannose 3,5‐epimerase	29	*Ct*‐miR319
Phosphomannomutase	24	–
α‐galactosidase	31	–
β‐galactosidase	150	–
UDP‐Galactose transporter	4	*Ct*‐miR399
Galactokinase	6	–
UDP‐Glycosyltransferase	31	*Ct*‐miR3066 (Novel)
UDP‐Glucosyltransferase	70	*Ct*‐miR3066 (Novel)
Sugar transporter	124	*Ct*‐ miR399 (Known)
Fructokinase	40	*Ct*‐ miR156 (Known)
GDP‐mannose 3,5 epimerase	3	*Ct*‐ miR319 (Known)
β‐glucosidase	126	*Ct*‐miR166 (Known)

### RT‐PCR of potential miRNAs in clusterbean

**Figure 10:**
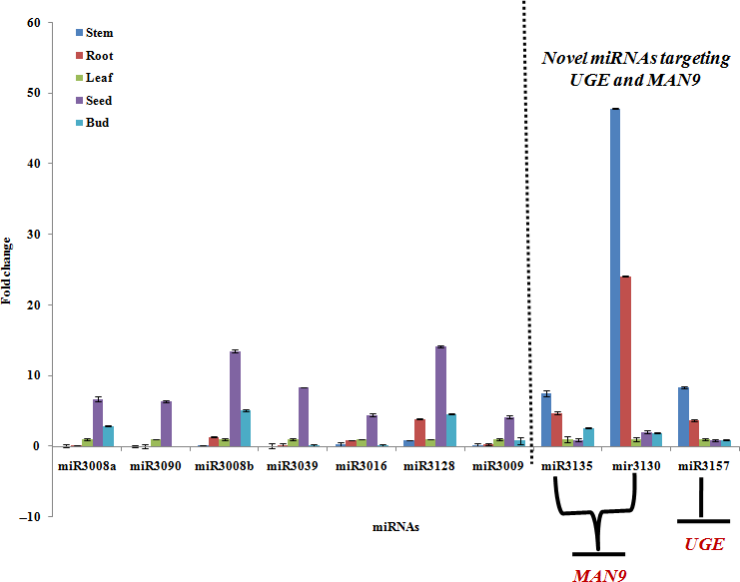
Relative expression analysis of 10 miRNAs, including nine novel miRNAs, *Ct*‐miR3008a, *Ct*‐miR3090, *Ct*‐miR3008b, *Ct*‐miR3039, *Ct*‐miR3016, *Ct*‐miR3128, *Ct*‐miR3130, *Ct*‐miR3135 and *Ct*‐miR3157 and one known miRNA,* Ct*‐miR3009 (miRNA156), based on qRT‐PCR in five different clusterbean tissues.

**Figure 11:**
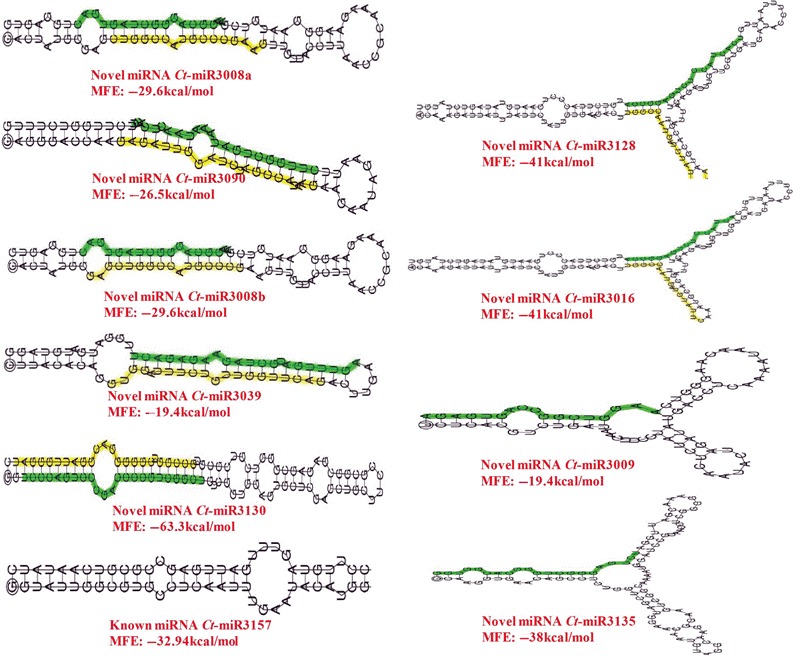
Secondary structures of the validated pre‐miRNAs (Mfold V3.2) showing the length and sequence details. miRNAs with star sequences are highlighted in yellow. Novel miRNAs are highlighted in red.

The expression levels of miRNAs, such as *Ct*‐miR3008a, *Ct*‐miR3090, *Ct*‐miR3008b, *Ct*‐miR3039, *Ct*‐miR3016, *Ct* miR3128, *Ct*‐mir3009, were found to be 6.68‐fold, 6.3‐fold, 13.48‐fold, 8.3‐fold, 4.4‐fold, 14.12‐fold and 4.2‐fold, respectively (Figure 10 and 11), showing comparatively higher expression in the seed than the control, that is leaf. Remarkably, among these miRNAs, the expression level of some miRNAs, such as *C*t‐miR3135, *Ct*‐miR3130 and *Ct*‐miR3157, was relatively higher in vegetative tissues (compared with reproductive tissues) with fold expression of 7.5, 47.8 and 8.37, respectively (Figure 10).

### Validation of miRNA targets using regional amplification quantitative RT‐PCR (RA‐RT‐PCR)

**Figure 12:**
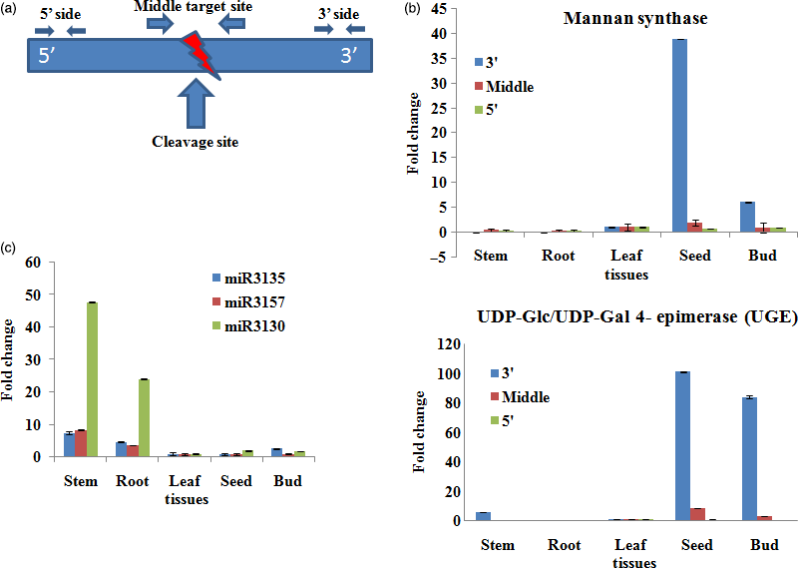
Regional amplification quantitative RT‐PCR (RA‐RT‐PCR) of the targets for five tissues (root, stem, leaf, bud and seed) in clusterbean. (a) Diagram of the primers designed to amplify the fragments of cleaved and noncleaved miRNA‐target sites. (b) Relative quantification of the three fragments of the two target genes (MAN9 and UGE) by RA‐PCR with the middle target fragment being set to a value of 1. (c) Relative expression of miRNA targets involved in the galactomannan pathway (*Ct‐*miR3135, *Ct‐*miR3157 and *Ct‐*miR3130) calculated by real‐time PCR with tubulin as the standard. Data are the means ± SE of three separate measurements.

As our attention was mainly on the galactomannan pathway, we further quantified and authenticated the inverse correlationship between the three miRNAs, viz. *Ct*‐mir3130, *Ct*‐miR3135 and *Ct*‐miR3157 and their potential target mRNAs through RA‐RT‐PCR, which is as good as 5′ RLM‐RACE (Table 3). Tissue‐specific as well as differential regulation of three miRNAs, that is *C*t‐miR3135, *Ct*‐miR3130 and *Ct*‐miR3157 and their corresponding targets MAN9 and UGE showed an inverse relationship, suggesting the specificity of these miRNAs towards these target genes (Figure 12).

## Discussion

Typically, miRNAs are known to negatively regulate the expression of genes at the post‐transcriptional level by binding to their mRNA targets via the RISC complex, leading to major variations in the activities of metabolic pathways (Gupta *et al*., 2017; Wu *et al*., 2016). They are known to regulate cell signalling (Yang *et al*., 2007), oxidative stress (Sunkar *et al*., 2006), abiotic and biotic stress responses (Khraiwesh *et al*., 2012), and the development of different tissues, including leaf, stem, root (Kidner and Martienssen, 2005), anther (Millar and Gubler, 2005) and flower (Yang *et al*., 2007). The conserved nature of plant miRNAs provides an opportunity to find homologous sequences in the nucleotide resources of unexplored plant species using *in silico* homology‐based approach (Das *et al*., 2012). Microarrays and high‐throughput sequencing data are the promising approaches that are widely used for the discovery of miRNAs, with the latter considered to be more effective method to discover miRNAs (Lee *et al*., 2002; Liu *et al*., 2008; Ricardi *et al*., 2014; Zuker, 2003).

Clusterbean is one such important galactomannan‐rich legume crop, having limited genomic and transcriptome resources. The galactomannans are known to be present at high levels within the endosperm of clusterbean seeds. To date, miRNA‐based regulation of glucomannan/galactomannan is reported only in *Amorphophallus* (Araceae) especially in leaves (Diao *et al*., 2014). Hence, our present investigation targets at generating knowledge about miRNA regulatory gene networks and metabolic processes that may influence galactomannan content of cultivated genotypes of *C. tetragonoloba*.

A comprehensive genome‐wide study was conducted in clusterbean for understating miRNAs‐based regulation of glucomannan biosynthesis in both vegetative as well as reproductive tissues. The genomes of closely related species as substitute references can assist in miRNA identification and expression studies in nonmodel species with no genome information (Etebari and Asgari, 2014). Therefore, cross‐species mapping of smallRNAseq data with closely related species, such as *G. max,* is a good opportunity for the identification and characterization of known and novel gene families. For dealing with known and novel miRNA identification in an unbiased way, two programs, viz. miRanalyzer and DSAP, were used that were already endorsed using *Aedes aegypti* (a nonmodel mosquito) as test with three well annotated insect genomes as proxy references. On the same fundamental and with same mapping criteria, a cross‐species mapping of the clusterbean miRNAs onto the *G. ma*x reference genome (as a proxy) was done, resulting in mapping of ~50%–68% reads (Table 2). As the *C. tetragonoloba* full‐genome sequence is not yet available, it is not possible to determine the accurate genomic locus of each miRNA family. The average length of the known precursors in the clusterbean ranged from 62 to 261 nt, whereas for novel miRNAs, it varied from 61 to 259 nt, in accordance with those previously reported in soybean (55–239 nt) and peanut (75–343 nt) (Chi *et al*., 2011). Here, the stringent minimum free energy (MFE) values further added confidence to the predicted hairpin structures as putative miRNA precursors. Interestingly, a higher number of known miRNAs were observed in the leaf (146) followed by the stem (134), root (104), seed (78) and bud (46) suggesting their conserved tissue‐specific as well as development‐specific roles. The counts of novel miRNAs in these tissues have shown the same pattern, viz. 87 miRNAs in the leaf, 83 in the stem, 47 in the root, 41 in the seed and 35 in the bud tissue (Tables S3 and S4).

It is likely that the *C. tetragonoloba* genome contains more miRNA‐encoding loci than Arabidopsis and rice, the genomes of which are fully sequenced. In Arabidopsis, 191 miRNA‐encoding loci (Nakano *et al*., 2006) and in rice 372 miRNA‐encoding loci (Nobuta *et al*., 2007) have been identified. Comparing these two libraries suggested that the bigger the genome, the more miRNA‐encoding loci there are.

In the present study, hierarchical clustering of the known and novel differential miRNAs has shown their existence within the same cluster and thus they can be hypothesized to follow functional co‐adaptation model exerting mutual repressive effects on target genes (Wang *et al*., 2016). Among known miRNA families, miRNA156, miR166 and miR1507 showed higher expression (Table S11), which are known to be highly conserved in many plant species, such as *Arabidopsis thaliana*,* Oryza sativa* and *Zea mays,* suggesting their involvement in conserved functions (Zhang *et al*., 2006). However, among the novel category miRNAs, *Ct*‐miRNA‐3008a, *Ct*‐miRNA‐3008b, *Ct*‐miRNA‐3090, *Ct*‐miRNA‐3039, *Ct*‐miRNA‐3016, *Ct*‐miRNA‐3132, *Ct*‐miRNA‐3066 and *Ct*‐miRNA‐3009, were highly expressed, signifying the evolution of these miRNAs for specialized function in clusterbean.

Transcription factors (TFs) and miRNAs are both considered as key regulators and have been well characterized in controlling the transcriptional events and ultimately the plant metabolic process (Djami‐Tchatchou *et al*., 2017; Nigam *et al*., 2015). TFs regulate gene expression by translating cis‐regulatory codes into specific gene‐regulatory events (Rothenberg, 2016). As TFs and miRNAs are both categorized as gene‐regulatory molecules and share a common regulatory logic (Hobert, 2008), they are proficient in cooperatively regulating the same gene: TFs regulate a gene's transcription in the gene's promoter region, while miRNAs regulate a gene's post‐transcription in the gene's 3′ untranslated region (UTR) and their respective targets form interconnected feedback and feed‐forward circuits (functional network) that are important for the execution of any metabolic process (Martinez and Walhout, 2009). In present study, enrichment of some miRNA‐targeted TFs, such as bHLH, NAC, bZIP, MYB64 and MYB83, implicates their involvement in cellulose synthase‐like A9 (CSLA9) expression, which is a crucial factor controlling galactomannan synthesis. Remarkably, a substantial number of TFs with multiple connections to specific miRNAs and other genes involved in the biosynthesis of carbohydrates further implies that these subset of TF, miRNAs and genes might synergistically regulate the galactomannan biosynthesis. In addition, the presence of three common TFs such as NAC, MYB and bHLH, among the known and novel miRNA hub communities, further proves their synergetic role/or co‐expression during galactomannan synthesis (Hu *et al*., 2017).

CSLA9 is responsible for the majority of glucomannan synthesis in both the primary and secondary walls of the Arabidopsis inflorescence stems (Goubet *et al*., 2009; Joet *et al*., 2014; Liepman *et al*., 2005). Both *in vitro* (EMSA) and *in vivo* (ChIP) binding assays clearly showed that MYB46 binds to the promoter of CSLA9 and its overexpression of MYB46 resulted in a significant increase in the mannan content (Kim *et al*., 2014). Moreover, physical interaction and regulatory synergy between particular subclasses of the MYB and bHLH (basic helix‐loop‐helix) TFs is well known in plant gene regulation (Du *et al*., 2009). Recent studies have shown that MYB46 function as a key regulator in the secondary wall formation, where it directly regulates the biosynthesis of the genes involved in three major components (i.e., cellulose, hemicellulose and lignin) of secondary walls (Ko *et al*., 2014). Therefore, MYB46 may be used for pathway‐specific manipulation of galactomannan biosynthesis. It was observed that these TFs and known miRNAs are densely coupled compared to the novel miRNAs, where they regulate each other or may target the same genes resulting in different combination of transcriptional/post‐transcriptional feed‐forward loops (FFLs) (Shalgi *et al*., 2007; Tsang *et al*., 2007). Moreover, the network‐based topological differences among the known and novel miRNAs and their common participation towards 35% of their targets already belonging to known miRNAs implied their spontaneous functional evolution and expansion in the clusterbean based on the functional requirement.

The strength of our study is that we have simultaneously used two deep‐sequencing RNA‐Seq data, that is miRNAseq and mRNAseq to survey paired changes in the miRNA and mRNA targets. Gene ontology results of the identified unigene targets reveal the association of the miRNA with carbohydrate metabolism, more specifically with the galactomannan pathway. A total of 22 enzyme families were identified (Table 3), and a model was constructed for the probable biosynthetic pathway of galactomannan in the clusterbean (Figure 9). The model predicts that galactomannan/glucomannan biosynthesis is related to sucrose metabolism, where sucrose (synthesized by photosynthesis during daytime) entering the developing seed is used to produce starch or galactomannan and other polysaccharides used by sucrose synthase (SuS) to produce fructose (Fru) and UDP‐Glc (Figure 9). Seven enzymes, namely sucrose synthase (SuS), phosphoglucose isomerase (PGI), phosphoglucomutase (PGM), phosphomannose isomerase (PMI), phosphomannomutase (PMM), starch synthase (SS) and UDP‐Glc/UDP‐Gal 4‐epimerase (UGE), were found in the clusterbean unigene data sets. Experimental evidence has also shown that these enzymes exhibit corresponding catalytic functions (Diao *et al*., 2014). Thus, the combinatorial analysis of small RNA and transcriptome data has the potential to identify regulatory miRNAs involved in biosynthetic pathway of galactomannan and the corresponding mRNA targets.

Earlier studies have focused on the miRNAs–mRNAs regulation pattern that includes both the coherent and incoherent relationships (Jeon *et al*., 2010; Zhao *et al*., 2013). To better understand the mode of miRNA regulation within the five tissues of the clusterbean, 18 miRNAs (of 25 densely correlated cluster) were picked (12 from the reproductive and six from vegetative tissue; Figure 10), analysed and validated. Interestingly, the expression profiles of 10 (of 18) mature miRNAs were found to be consistent with the results obtained by small RNA deep sequencing, indicating that the sequencing data produced in the present study is reliable and could be subjected to further analysis.

In addition, quantitative RT‐PCR (qRT‐PCR) for nine novel miRNAs (*Ct*‐miR3008a, *Ct*‐miR3090, *Ct*‐miR3008b, *Ct*‐miR3039, *Ct*‐miR3016, *Ct*‐miR3128,*Ct*‐miR3130, *Ct*‐miR3135 and *Ct*‐miR3157) and one known miRNA, that is *Ct*‐miR3009 (miR156 family), showed differential expression schema (Figure 10). Among these, the expression pattern of some miRNAs, viz. *Ct*‐miR3130, *Ct*‐miR3135 and *Ct*‐miR3157, revealed tissue‐specific differential regulation as well as negative correlation with their targets (Millar and Gubler, 2005; Yang *et al*., 2007). Our regional amplification quantitative RT‐PCR (RA‐RT‐PCR) assay further established the cleavage specificity of these miRNAs towards their target transcripts (Oh *et al*., 2008) (Figure 12). Apparently, in contrast to these miRNAs, expression pattern of their targets, that is MAN9 and UGE (known to be involved in the galactomannan pathway), was in opposite order showing higher expression in seed and bud tissue (but insignificant in vegetative tissues), viz. 38.9‐ and 101.4‐fold, 5.9 and 84.3‐fold, respectively (Figure 12b,c). In agreement with the previous studies, our results also showed the lower accumulation of RA‐Mid region than the RA 3′ region signifying that these target genes are potentially cleaved by corresponding regulatory miRNAs (Oh *et al*., 2008; Gupta *et al*., 2017).

Our combinatorial approach of fusing two different miRNA identification softwares with two types of deep‐sequencing data from same platform in clusterbean provides a systematic overview on novel and known miRNAs expression profiling involved in galactomannan biosynthesis. These candidate miRNAs and their target genes from vegetative and reproductive tissues would enhance our understanding of the miRNA regulatory tissue‐specific metabolic pathways in clusterbean. In conclusion, this is the first comprehensive report on the identification and characterization of miRNAs and their potential targets postulated to act in the galactomannan biosynthesis pathway of clusterbean.

## Experimental procedures

### Plant materials

A total of five clusterbean (cultivar RGC 936) tissues, viz. root, stem, leaf, bud and seed were collected from the Phenotyping Facility, NRCPB, New Delhi, fixed in liquid nitrogen and stored at −80 °C (Figure 1). Total RNA from each sample was isolated using TRIzol reagent as per the manufacturer's instruction (Thermo Fisher Scientific, Waltham, MA, USA). The quality and quantity of each total RNA samples were checked on denaturing agarose gels and Nanodrop (Thermo, Fisher Scientific, Wilmington, DE, USA), respectively.

### Small RNA library preparation

Small RNA libraries were prepared using isolated total RNA according to the protocol of the Illumina TrueSeq Small RNA Library Preparation Kit (https://support.illumina.com/). The protocol includes adapter ligation, reverse transcription, PCR amplification and pooled gel purification. The selected gel size and purified libraries were analysed using the Agilent 4200 Tape station system (Agilent Technologies, Santa Clara, CA, USA) with high sensitivity D1000 Screen tape as per the manufacturer's instruction. After obtaining the Qubit concentration for the libraries and the mean peak size from the Agilent tape station profile, the SE Illumina libraries were loaded onto NextSeq 500 for cluster generation and sequencing.

### *In silico* analysis of miRNAs and target prediction

Raw data were subjected to adapter filtration Trimmomatic version 0.36 (https://github.com/timflutre/trimmomatic.git) at default parameters. Quality trimming and adaptor removal of the reads were performed by Trimmomatic (v 0.32) with default parameters ‘LEADING:5, TRAILING:5, MINLEN:36′ (Bolger *et al*., 2014). A Phred quality score of 30 was used to trim the terminal low‐quality bases, and reads <15 bp were discarded and >30 bp were trimmed off. Unique contigs and their counts were generated for the individual samples using an in‐house script. Small RNA sequencing data are known to include reads from known small noncoding RNAs, such as tRNAs, snoRNAs, piRNAs and snRNAs. To remove these, we blasted read contigs against the customized Rfam database (v12.2.) (http://rfam.xfam.org/). The reads aligned onto the Rfam database, and those satisfying the above‐mentioned criteria were filtered out; then, filtered reads were again mapped to known repeat sequences, that is Repbase (v 21) (http://www.girinst.org/repbase/) using Blastn (with same parameters), and subsequently removed. A tab‐separated file with the read sequences and its counts from all five tissues was used as the input file for miRanalyzer (Hackenberg *et al*., 2011) and DSAP (Huang *et al*., 2010). An updated version of miRanalyzer, a web‐based server (https://github.com/shenlab sinai/miRNA_pipeline_for_miRanalyzer), was used as the main pipeline for the detection of known miRNAs and the prediction of novel miRNAs. This software is based on a random forest classifier and implements an extremely accurate machine learning algorithm (Support Vector Machine) to predict new miRNA candidates from high‐throughput sequencing data (Hackenberg *et al*., 2009). Bowtie, an ultrafast short read aligner, was used to map the reads to the *G. max* (as proxy) genomes and miRNA database (miRBase v.21; http://www.mirbase.org). Further, DSAP (deep‐sequencing small RNA analysis pipeline; http://dsap.cgu.edu.tw.) was also used as a control to enhance our confidence to eliminate the impact of the software performance on data analysis. For the discovery of known miRNAs, the clustered reads were mapped with a nonredundant mature miRNA reference of *G. max*, using word‐match and the Smith–Waterman algorithm (Li *et al*., 2012).

Those sequences that were unmapped against *G. max* (proxy genome) were further fed to triplet‐SVM (http://bioinfo.au.tsinghua.edu.cn/mirnasvm/) for the identification of potential novel miRNAs. Stem‐loop hairpin secondary structures of these known and novel miRNAs were predicted via RNAfold software (http://rna.tbi.univie.ac.at/cgi-bin/RNAfold.cgi) and RNAPlot (https://www.tbi.univie.ac.at/RNA/RNAplot.1.html) in the background. The criteria for secondary structure prediction were as described by previous studies (Schmittgen and Livak, 2008).

### miRNA‐target identification

PsRNATarget (http://bioinfo3.noble.org/psRNATarget/) and psRobot (http://omicslab.genetics.ac.cn/psRobot/) both were used to identify target transcripts using the mechanism for analysing complementary matching between the sRNA and targets by evaluating target site accessibility through calculating the binding energy using Smith–Waterman algorithm. The *G. max* unigenes (JGI genomic project, Phytozome), as well as a recently published high‐quality 48 007 clusterbean unigenes (FPKM > 1 and length ≥ 200 bp) (Rawal *et al*., 2017), were used as a target data set using the default parameters. Firstly, the CDS (coding sequence) from these unigenes were extracted using TransDecoder software (https://github.com/TransDecoder/TransDecoder) and then used for subsequent analysis. Comparisons/overlaps were made for common target predictions among the both algorithms. The detailed target annotations were obtained from the Uniprot database of Viridiplantae (e‐value 10^−7^) using Blast2GO software. Enrichment analyses of the identified miRNA targets (based on Fisher's exact test) were performed with a singular enrichment analysis (SEA) method available within AgriGO tool v2.0 (http://bioinfo.cau.edu.cn/agriGO/).

### Differential expression analysis

After aligning onto *G. max* as a reference genome using miRanalyzer, read counts were used for expression profiling of known and novel miRNAs separately in all the possible combinations. RKPM (reads per kilobase million per mapped reads) normalization was applied to normalize the read count to the length of the miRNA. RKPM values were then log (base 2)‐transformed to get the fold expression values calculated by subtracting the respective fold expression values. Fold change values of >1 were considered up‐regulated, whereas those <‐1 were considered to be down‐regulated. A Multi Experiment Viewer, TM4, Microarray suit, Boston, MA, USA (MeV, v4.7.4) (Saeed *et al*., 2006) software tool was used to produce the hierarchical clustering of the different tissues, based on the K‐means method (Sturn *et al*., 2002) and using the Euclidean distance and average linkage method.

### Conserved miRNA sequence multiple alignments and phylogenetic analysis

Conserved miRNA multiple alignments were performed using WebLogo at the URL: http://weblogo.berkeley.edu/logo.cgi. Because most plant miRNAs and their precursor sequences are derived from the same gene families, they are strongly conserved and have high sequence identity, even between distantly related species. To determine the phylogeny of the clusterbean miRNAs, we derived all the precursor sequences of three closely related plant species, including *C. cajan, G. max* and *M. truncatula,* from miRBase release 21. These precursor sequences, including miRNA clusterbean precursors, were subjected to multiple sequence alignment MEGA version 5.0, Tempe, Arizona (Tamura *et al*., 2011). Bootstrap confidence values were obtained by applying 1000 replications. Moreover, to determine the correlations among miRNAs and miRNA‐target clusterbean genes, an interactome network was constructed according to the Cytoscape software manual (http://www.cytoscape.org).

### Expression analysis of clusterbean miRNAs using qRT‐PCR

Small RNAs (1 μg) were isolated from five different plant tissues, including the bud, leaf, root, stem and seed, poly‐adenylated and reverse‐transcribed with Mir‐X miRNA First‐Strand Synthesis kit (Clontech, CA, USA) following the manufacturer's instructions. The reaction (1× mRQ buffer and 1 μL of mRQ enzyme mix) was terminated after 1 h by incubation at 85 °C for 5 min. For detection of the predicted miRNAs, the cDNAs from the previous step were diluted 20 times in RNase‐free water, and 2 μL of the diluted cDNA was used in a total reaction volume of 25 μL, which was amplified using a C1000 touch thermal cycler Bio‐Rad PCR system using predicted mature miRNA sequence as the sense (forward) primer and mRQ 3′ primer provided with the Mir‐X miRNA qRT‐PCR SYBR kit (Clontech, CA, USA) as the antisense primer (reverse). The PCR products were run on a 4% agarose gel for desired band confirmation. After confirming the sequences of the amplified PCR products, qRT‐PCR analysis was performed in a 96‐well plate using Roche 454 qPCR system (Roche, Branford, Ct, USA). Briefly, PCR mixtures of 25 μL were prepared with 1× SYBR Advantage Premix, 1× ROX dye, 0.2 μm each of the forward and reverse primers as indicated above and 2 μL of the first‐strand cDNA. The reactions were conducted in a 96‐well PCR plate at 95 °C for 2 min, followed by 40 cycles of 95 °C for 10 s and 60 °C for 20 s. The melting curves of the cycles were analysed from 56 °C to 95 °C, with an increment of 0.5 °C every 10 s. To establish the relative expression levels of each sample, the ‘comparative Ct method’ (Schmittgen and Livak, 2008) was used. The threshold cycle (Ct) value of each individual reaction was normalized to the Ct value of the U6 snRNA (U6 snRNA primer was provided with the kit) whose expression was found to be consistent across the conditions. All the reactions were conducted in three technical replicates. All the 10 forward miRNA‐specific primers, which were used to amplify the corresponding miRNAs along with the gene‐specific primers of their targets for qRT‐PCR analysis, are listed in Table 4. These mature miRNA sequence are present in the 5′ arm having a precursor length ranging from 69 to 138 as shown in (Table 4). U6 (Ubiquitin 6) was used for each sample as an endogenous control. Structures of these validated miRNAs are shown in Figure 11.

**Table 4:** The primers list used for mature miRNA validation using qRT‐PCR

Novel miRNA	Sequence	Arm	Orientation	MFE (kcal/mol)	Length (nt)	Precursor length	Precursor coordinates	Pu/Py rich
*Ct*_ miR 3008aF	AAGGCAGGGCTAGTGAC **mRQ 3′ primer** a	5′	Negative strand	−29.6	17	83	44580810–44580892	PU rich
*Ct*_ miR 3090F	CTTGGCTGATAAATAACTCACT **mRQ 3′ primer** a	5′	‐do‐	−26.5	22	77	14962548–14962569	PY rich
*Ct*_ miR 3008bF	AAGGCAGGGCTAGTGAC **mRQ 3′ primer** a	5′	‐do‐	−29.6	17	83	44580818–44580838	PU rich
*Ct*_ miR 3039F	AGTTTGATGCTAGAAGAGACT **mRQ 3′ primer** a	5′	‐do‐	−19.4	21	69	37897185–37897205	PU rich
*Ct*_ miR 3128F	TCACTACCTCTGAGGCCA **mRQ 3′ primer** a	5′	‐do‐	−41	18	138	2231912–2232049	PU rich
*Ct*_ miR 3016F	ACTACCTCTGAGGCCA **mRQ 3′ primer** a	5′	‐do‐	−41	16	138	2231912–2232049	PY rich
*Ct*_ mir 3130F (Known)	TCCCAGTCCCGACCCCGTCGGCT **mRQ 3′ primer** a	5′	‐do‐	−63.3	23	99	3380503–3380601	PU rich
*Ct*_ miR 3009F	TGACAGAAGAGAGTGAGCAC **mRQ 3′ primer** a	5′	‐do‐	−48	20	83	5047203–5047285	PU rich
*Ct*_ miR 3135F	TCTGAGGGCTGGGCACGGTGG **mRQ 3′ primer** a	5′	‐do‐	−38	21	107	5137839–5137859	PU rich
*Ct*_ miR 3157F	TTGAGCCGCGTCAATATCT **mRQ 3′ primer** a	5′	Positive strand	−51.7	19	80	35464073–35464091	PU rich

a mRQ3′ primer was provided by the Mir‐X™ miRNA First‐Strand Synthesis and SYBR® qRT‐PCR kit (Clonetech, CA, USA).

### Validation of predicted miRNA‐target gene by regional amplification quantitative RT‐PCR (RA‐RT‐PCR)

To further quantify and validate the expression levels of the predicted target transcripts of the galactomannan pathway‐related clusterbean miRNA, regional amplification quantitative RT‐PCR (RA‐PCR) test was performed. Total 2 target transcripts were derived from the clusterbean unigenes (Rawal *et al*., 2017). The RA‐PCR was developed to inspect the miRNA‐directed cleavage of mRNAs (Oh *et al*., 2008). MicroRNA‐mediated cleavage of the target site of the mRNA transcripts leads to a decline in the RT‐PCR accumulation of any fragment present upstream of the target site (Navarro *et al*., 2006). As reverse transcription of the miRNA‐targeted mRNAs will not produce a cDNA beyond the cleaved site, the segment of cDNA present upstream of the cleaved site can be expected to be amplified in lesser quantity than a segment existing downstream of the cleaved site. RA‐PCR has further added advantage, including a control reaction in the same experiment, while other methods, such as 5′ rapid amplification of cDNA ends (RACE) (5′ RACE), require separate reactions and could be skewed by the existence of false positives. IDT (Integrated DNA Technologies Inc., San Diego, CA, USA) software was used to design the specific primers for quantitative RT‐PCR (Table 5), and qPCR was performed using the Roche 454 qPCR system (Roche, Branford, USA) with SYBR® Green JumpStart™ (SIGMA St. Louis, MO, USA). Briefly, each 10 μL PCR contained ~100 ng cDNA, 7 μL 2× SYBR Green JumpStart Taq Ready Mix and 200 nm of each primer. The PCR conditions were as follows: 95 °C for 2 min, followed by 40 cycles of 95 °C for 10 s and 60 °C for 30 s. A final ramping stage of 56–95 °C was performed to confirm the absence of multiple products and primer dimers. Tubulin was used for each sample as an endogenous control. All samples were subjected to at least three technical replicates. The data were analysed using the Ct (2^−ΔΔCt^) method described above. For each potential mRNA target transcript, a total of three sets of primers were designed to amplify three different regions (5′ region, middle region and 3′ region).

**Table 5:** List of the target primers used in the RA‐RT‐PCR validation

S.No.	miRNA	Target	Target total length (bp)	Primer name	Primer region (bp)	F Primer (5′**–**3′)	R Primer (5′**–**3′)	Amplicon size (bp)	*T*m (^**°**^C)
1.	*Ct*‐mir 3130	MAN9	1857	RA‐Mid	80–185	TGCTTGGACTTACCTGCTTATT	AATAATCTCCTCCCGCAGAAAG	105	62
				RA‐5′	80–185	TGCTTGGACTTACCTGCTTATT	AATAATCTCCTCCCGCAGAAAG	105	62
				RA‐3′	119–242	AATAATCTCCTCCCGCAGAAAG	AATAATCTCCTCCCGCAGAAAG	123	62
2.	*Ct*‐miR 3135	MAN9	1857	RA‐Mid	80–185	TGCTTGGACTTACCTGCTTATT	AATAATCTCCTCCCGCAGAAAG	105	62
				RA‐5′	80–185	TGCTTGGACTTACCTGCTTATT	AATAATCTCCTCCCGCAGAAAG	105	62
				RA‐3′	119–242	AATAATCTCCTCCCGCAGAAAG	AATAATCTCCTCCCGCAGAAAG	123	62
3.	*Ct*‐miR 3157	UGE	2657	RA‐Mid	465–576	CATGGAGAATCCTGGCTCTTA	CTACTAGAGGCCCACACAATAG	111	61
				RA‐5′	469–569	GAGACCCATACCTCCAAGAAAC	GACTCGGTTTGGTGAGTTAGTT	100	62
				RA‐3′	115–234	GGGAAGAAAGCTGTTAGTGA	GTGATCGTACAACGAACAGAGA	119	62

Internal control genes					
S. No.	Gene	F Primer (5′–3′)	R Primer (5′–3′)	Amplicon size (bp)	*T*m (°C)
1.	Tubulin	GAGTCCCCACCATACCTTCC	TGCGTGATCCTTCACATC	117	60

## Conflict of interest

The authors declare no competing financial interests.

## Supporting information


**Figure S1** Major functional classification observed within miRNA targeted Clusterbean unigenes using Blast2GO annotation.

**Table S1** A brief overview of the leguminous and non‐leguminous plants possessing galactomannan.

**Table S2** Number of precursor and mature miRNAs reported already for the nine species of Leguminosae families.

**Table S3** Counts of known miRNAs identified commonly through miRanalyzer and DSAP software in five different tissues of clusterbean, including the bud (Ct‐B; Sheet 1), leaf (Ct‐L; Sheet2), shoot (Ct‐S; Sheet3), root (Ct‐R; Sheet3) and stem (Ct‐St; Sheet3).

**Table S4** Counts of novel miRNAs identified commonly through miRanalyzer and DSAP software in five different clusterbean tissues, including the bud (Ct‐B; Sheet 1), leaf (Ct‐L; Sheet2), shoot (Ct‐S; Sheet3), root (Ct‐R; Sheet3) and stem (Ct‐St; Sheet3).

**Table S5** Targets (using *Glycine max* unigenes) identified using the PsRNATarget and psRobot tool for known miRNAs (in pooled form) in five different clusterbean tissues.

**Table S6** Targets (using *Glycine max* unigenes) identified using the PsRNATarget and psRobot tool for novel miRNAs (in pooled form) in five different clusterbean tissues.

**Table S7** Known and novel miRNAs targets (in pooled form) identified from the Unigenes of the clusterbean (after pooled assembly of transcriptome data derived from three tissues, i.e., Leaf, Flower and Shoot) using the PsRNATarget and psRobot tool.

**Table S8** Biological processes (BPs) based on singular enrichment analysis (SEA) for miRNA targeted clusterbean unigenes. The difference between the query and background ratio of a particular Gene Ontology term are shown in different colors. A Fisher's exact test with FDR corrected *P* value of <0.05 was used as a parameter.

**Table S9** Molecular Functions (MFs) based on singular enrichment analysis (SEA) for miRNA targeted clusterbean unigenes. The difference between the query and background ratio of a particular Gene Ontology term are shown in different colors. A Fisher's exact test with FDR corrected *P* value of < 0.05 was used as a parameter.

**Table S10** Cellular Components (CCs) based on singular enrichment analysis (SEA) for miRNA targeted clusterbean unigenes. The difference between the query and background ratio of a particular Gene Ontology term are shown in different colors.

**Table S11** Differentially expressed known miRNAs commonly identified from miRanalyzer and DSAP software within five different clusterbean tissues, including bud (Ct‐B; Sheet 1), leaf (Ct‐L; Sheet2), shoot (Ct‐S; Sheet3), root (Ct‐R; Sheet3) and stem (Ct‐St; Sheet3). Here, a fold change of ≥1 and <−1 was used with a *P* value < 0.05.

**Table S12** Differentially expressed novel miRNAs commonly identified from miRanalyzer and DSAP software within five different clusterbean tissues, including bud (Ct‐B; Sheet 1), leaf (Ct‐L; Sheet2), shoot (Ct‐S; Sheet3), root (Ct‐R; Sheet3) and stem (Ct‐St; Sheet3). Here, a fold change of ≥1 and <−1 was used with a *P* value < 0.05.

**Table S13** Different enzyme classes observed within the miRNA targeted clusterbean unigenes using Blast2GO annotation.
