# Supplementary figures and images for: Genome‐wide discovery of tissue‐specific miRNAs in clusterbean (Cyamopsis tetragonoloba) indicates their association with galactomannan biosynthesis
Source: Plant Biotechnol J. 2018 Mar 11;16(6):1241–57. doi: 10.1111/pbi.12866 (PMC5978871; doi:10.1111/pbi.12866)

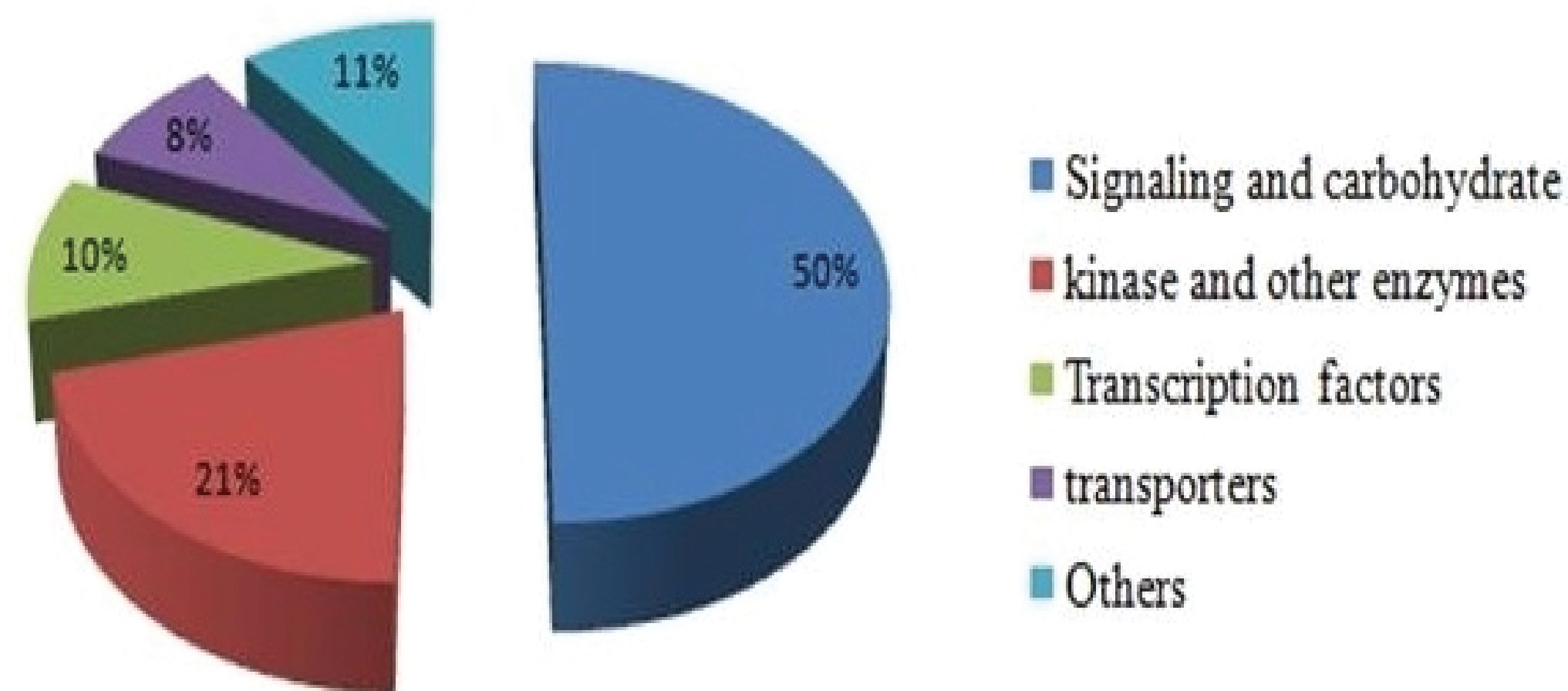

Supplement: Supplementary file 1 — Figure S1 Major functional classification observed within miRNA targeted Clusterbean unigenes using Blast2GO annotation. [file PBI-16-1241-s014.jpg]
