# Supplementary material for: Genome‐wide discovery of tissue‐specific miRNAs in clusterbean (Cyamopsis tetragonoloba) indicates their association with galactomannan biosynthesis
Source: Plant Biotechnol J. 2018 Mar 11;16(6):1241–57. doi: 10.1111/pbi.12866 (PMC5978871; doi:10.1111/pbi.12866)
Supplement: Supplementary file 2 — Table S1 A brief overview of the leguminous and non‐leguminous plants possessing galactomannan. [file PBI-16-1241-s012.docx]

**Table S1: Galactomannan content in leguminous and non-leguminous plant species**

| **Leguminous species** |  |  |  |
| --- | --- | --- | --- |
| **Subfamily** | **Botanical name** | **M/G ratio** | **Reference** |
| **CAESALPINIACAE** | *Cassia absus* | 3.00 | Aftab K. et al., 1996; Kapoor V. P. and Mukherjee S., 1971; Pandya H. et al., 2010 |
|  | *C. emarginata* | 2.7 | Dea I.C.M. and Morrison A., 1975 |
|  | *C. Fistula* | 3 | Gupta M. et al., 2000; Petkowicz C.L.O. et al., 1998 |
|  | *C. leptocarpa* | 3.05 | Sharma B.R. et al., 2008 |
|  | *C. marylandica* | 3.76 | Kapoor V.P., 1994 |
|  | *C. nodosa* | 2.7-3.5 | Prajapati V.D. et al., 2013 |
|  | *C. occidentalis* | 3 | Kapoor V.P., 1994 |
|  | *C. tora* | 3 | Pawar H. 2014; Shang M. 2012; Srivastava M. and Kapoor V.P., 2005 |
|  | *Ceratonia siliqua* | 3.75 | Dakia P.A. et al., 2008; Doyle J.P. et al., 2006; Prajapati V.D. et al., 2013; Prado B.M. et al., 2005 |
|  | *Caesalpinia cacalaco* | 2.5 | Srivastava M. and Kapoor V.P., 2005 |
|  | *C. pulcherima* | 2.7 | Cerqueira M.A. et al., 2009; Cerqueira M.A. et al., 2011 |
|  | *C. spinosa* | - | Srivastava M. and Kapoor V.P., 2005; Prado B.M. et al., 2005 |
|  | *Cercidium torreyanum* | 3.38 | Sharma B.R. et al., 2008 |
|  | *Delonix regia* | 3.28 | Dea. ICM and Morrison. A., 1975 |
|  | *Gleditsia amorphoides* | 2.5 | Perduca M.J. et al., 2013 |
|  | *G. triacanthos* | 3.2 | Bourbon A.I. et al., 2010; Cerqueira M.A. et al., 2009; Cerqueira M.A. et al., 2010; Srichamroen A. et al., 2009 |
|  | *Gymnocladus dioica* | 2.71 | Prajapati V.D. et al., 2013 |
|  | *Parkinsonia aculeata* | 2.70 | Prajapati V.D. et al., 2013 |
| **MIMOSACEAE** | *Besmanthus illinoensis* | 2.69 | Srivastava M. and Kapoor V.P., 2005 |
|  | *Leucaena galauca* | 1.33 | Srivastava M. and Kapoor V.P., 2005 |
| **FABACEAE** | *Sophora japonica* | 5.19 | Bourbon A.I. et al., 2010; Cerqueira M.A. et al., 2009 |
|  | *Genista raetam* | 4.14 | Sharma B.R. et al., 2008 |
|  | *G. scoparia* | 1.59 | Sharma B.R. et al., 2008 |
|  | *G. cretica* | 1.56-167 | Sharma B.R. et al., 2008 |
|  | *G. foenum-graecum* | 1.2 | Brummer Y. et al., 2003; Prado B.M. et al., 2005; Sindhu G. et al., 2012 |
|  | *G. hamosa* | 1.17 | Sharma B.R. et al., 2008 |
|  | *G. monspeliaca* | 1.08 | Sharma B.R. et al., 2008 |
|  | *G. polyserata* | 1.13 | Sharma B.R. et al. , 2008 |
|  | *G. radiata* | 1.17 | Sharma B.R. et al. , 2008 |
|  | *Anthyllis vulneraria* | 1.33 | Sharma B.R. et al. , 2008 |
|  | *Lotus corniculatus* | 1.25 | Sharma B.R. et al. , 2008 |
|  | *L. pedunculatus* | 1.04 | Sharma B.R. et al. , 2008 |
|  | *L. scoparius* | 1.13 | Sharma B.R. et al. , 2008 |
|  | *Alysicarpus veginalis* | 1.14 | Sharma B.R. et al. , 2008 |
|  | *Desmodium pulchellum* | 2.0 | Sharma B.R. et al. , 2008 |
| **Non leguminous species** |  |  |  |
| **ANNONACEAE** | *Annona muricata* | 4.46 | Sharma B.R. et al. , 2008 |
| **CONVOLVULACEAE** | *Convolvulus tricolor* | 1.75 | Sharma B.R. et al. , 2008 |
|  | *Ipomoea muricata* | 1.8 | Sharma B.R. et al. , 2008 |
| **EBENACEAE** | *Diospyros virginiana* | - | Sharma B.R. et al. , 2008 |
| **LOGANIACEAE** | *Strychnos nux-vomica* | - | Sharma B.R. et al. , 2008 |
| **PALMAE** | *Borassus flabellifer* | 2.4 | Sharma B.R. et al. , 2008 |
|  | *Cocos mucifera* | 2.57 | Sharma B.R. et al. , 2008 |
|  | *Arenga saccharifera* | 2.26 | Sharma B.R. et al. , 2008 |
|  | *Phytelephas macrocarpa* | 50 | Sharma B.R. et al. , 2008 |
|  | *Hyphaene thebaica* | 19 | Sharma B.R. et al. , 2008 |
|  | *Phoenix dactylifera* | 10 | Sharma B.R. et al. , 2008 |
