# Supplementary material for: Genome‐wide discovery of tissue‐specific miRNAs in clusterbean (Cyamopsis tetragonoloba) indicates their association with galactomannan biosynthesis
Source: Plant Biotechnol J. 2018 Mar 11;16(6):1241–57. doi: 10.1111/pbi.12866 (PMC5978871; doi:10.1111/pbi.12866)
Supplement: Supplementary file 3 — Table S2 Number of precursor and mature miRNAs reported already for the nine species of Leguminosae families. [file PBI-16-1241-s011.docx]

| Species | miRNA Precursors | mature miRNAs |
| --- | --- | --- |
| *Acacia auriculiformis* | 7 | 7 |
| *Arachis hypogaea* | 23 | 32 |
| *Acacia mangium* | 3 | 3 |
| *Glycine max* | 573 | 639 |
| *Glycine soja* | 13 | 13 |
| *Lotus japonicus* | 62 | 67 |
| *Medicago truncatula* | 672 | 756 |
| *Phaseolus vulgaris* | 8 | 10 |
| *Vigna unguiculata* | 18 | 18 |

**Table S2. Number of precursor and mature miRNAs reported already for nine species of Leguminosae families**
