# Supplementary material for: Genome‐wide discovery of tissue‐specific miRNAs in clusterbean (Cyamopsis tetragonoloba) indicates their association with galactomannan biosynthesis
Source: Plant Biotechnol J. 2018 Mar 11;16(6):1241–57. doi: 10.1111/pbi.12866 (PMC5978871; doi:10.1111/pbi.12866)
Supplement: Supplementary file 9 — Table S8 Biological processes (BPs) based on singular enrichment analysis (SEA) for miRNA targeted clusterbean unigenes. The difference between the query and background ratio of a particular Gene Ontology term are shown in different colors. A Fisher's exact test with FDR corrected P value of <0.05 was used as a parameter. [file PBI-16-1241-s005.pdf]

Term	Qu_ratio	BG_ratio	Ratio_Q/B
carbohydrate biosynthetic process	0.19963	0.01938	10.30066
carbohydrate catabolic process	0.139155	0.013893	10.01631
carbohydrate homeostasis	0.004937	0.000398	12.40749
carbohydrate mediated signaling	0.031163	0.003548	8.783805
carbohydrate metabolic process	0.580376	0.05965	9.729774
carbohydrate phosphorylation	0.003703	0.000298	12.40749
carbohydrate transport	0.058932	0.005388	10.93768
carbon fixation	0.018821	0.001674	11.24045
carbon utilization	0.005245	0.002172	2.415198
cell wall biogenesis	0.099044	0.008704	11.37944
cell wall glycoprotein biosynthetic process	0.001543	0.000166	9.305616
cell wall macromolecule biosynthetic process	0.017587	0.001492	11.78711
cell wall macromolecule catabolic process	0.016662	0.002072	8.040052
cell wall macromolecule metabolic process	0.048442	0.004675	10.36157
cell wall modification	0.07004	0.006316	11.08858
cell wall polysaccharide biosynthetic process	0.014193	0.001127	12.58995
cell wall polysaccharide metabolic process	0.029003	0.002321	12.49611
cell wall thickening	0.005554	0.000448	12.40749
cellular carbohydrate biosynthetic process	0.163838	0.015136	10.82428
cellular carbohydrate catabolic process	0.11046	0.010743	10.28213
cellular carbohydrate metabolic process	0.390003	0.033671	11.58277
cellular cell wall organization	0.034866	0.011754	2.966247
cellular cell wall organization or biogenesis	0.132984	0.021453	6.198949
cellular polysaccharide biosynthetic process	0.099352	0.009466	10.4953
cellular polysaccharide catabolic process	0.017279	0.001857	9.305616
cellular polysaccharide metabolic process	0.142549	0.01381	10.3222
cellular potassium ion homeostasis	0.023758	0.002321	10.23618
cellular respiration	0.061401	0.005222	11.75757
cellular response to chemical stimulus	0.510336	0.048111	10.6075
cellular response to extracellular stimulus	0.0722	0.00625	11.5518
cellular response to glucose starvation	0.00216	0.000216	10.02143
cellular response to heat	0.001851	0.000116	15.95248
cellular response to hormone stimulus	0.356989	0.035445	10.07165
cellular response to hydrogen peroxide	0.014502	0.001111	13.05564
cellular response to nitrogen levels	0.010799	0.000912	11.84351
cellular response to oxidative stress	0.020673	0.00131	15.78421
cellular response to phosphate starvation	0.026226	0.002288	11.46344
cellular response to reactive oxygen species	0.019438	0.001111	17.50011
cellular response to starvation	0.052144	0.004675	11.15354
cellular response to stimulus	0.790805	0.075698	10.44691
cellular response to stress	0.315643	0.028681	11.00537
cellular response to sucrose starvation	0.00216	0.000149	14.4754
cellular response to water deprivation	0.009565	0.000829	11.53896
cellular sodium ion homeostasis	0.004937	0.000564	8.758226
cellulose biosynthetic process	0.025918	0.002387	10.85655
cellulose catabolic process	0.007097	0.000713	9.954845

cellulose metabolic process	0.03178	0.002918	10.8918
cellulose microfibril organization	0.009565	0.000895	10.68423
chaperone mediated protein folding requiring cofactor	0.008331	0.000647	12.8847
chaperone-mediated protein complex assembly	0.00216	0.000149	14.4754
chlorophyll biosynthetic process	0.029312	0.002404	12.19357
chlorophyll catabolic process	0.006171	0.000746	8.271658
chlorophyll metabolic process	0.039494	0.003399	11.62067
chloroplast accumulation movement	0.00216	0.000414	5.211145
chloroplast avoidance movement	0.004937	0.00058	8.507991
chloroplast fission	0.005245	0.000812	6.456958
chloroplast organization	0.038568	0.003697	10.4323
chloroplast relocation	0.007714	0.000928	8.308585
chloroplast RNA processing	0.003394	0.000381	8.901024
chloroplast-nucleus signaling pathway	0.003085	0.000216	14.31633
chromatin assembly	0.044122	0.00431	10.23618
chromatin assembly or disassembly	0.059241	0.006283	9.428381
chromatin disassembly	0.003394	0.000265	12.79522
chromatin modification	0.101512	0.011754	8.636241
chromatin organization	0.157976	0.017905	8.823102
chromatin remodeling	0.030238	0.003299	9.16533
chromatin remodeling at centromere	0.00216	0.000149	14.4754
chromatin silencing	0.033323	0.00368	9.054112
chromatin silencing at centromere	0.004011	0.000414	9.67784
chromatin silencing at silent mating-type cassette	0.006788	0.000663	10.23618
chromatin silencing at telomere	0.005862	0.000497	11.78711
chromatin silencing by small RNA	0.004937	0.00063	7.836308
chromatin-mediated maintenance of transcription	0.00216	0.000282	7.663448
defense response	0.459118	0.054228	8.466375
defense response signaling pathway, resistance gene-depender	0.011725	0.001293	9.06701
defense response signaling pathway, resistance gene-independ	0.010182	0.00121	8.413296
defense response to bacterium	0.146868	0.014904	9.854222
defense response to bacterium, incompatible interaction	0.052144	0.004741	10.99755
defense response to fungus	0.120642	0.012848	9.389666
defense response to fungus, incompatible interaction	0.019747	0.00189	10.44841
defense response to insect	0.004937	0.00063	7.836308
defense response to oomycetes	0.006788	0.000647	10.49864
defense response to virus	0.031163	0.003183	9.790283
defense response, incompatible interaction	0.116322	0.012119	9.598405
fatty acid alpha-oxidation	0.001543	0.000199	7.75468
fatty acid beta-oxidation	0.026226	0.002089	12.5552
fatty acid biosynthetic process	0.11879	0.011257	10.55276
fatty acid catabolic process	0.02962	0.002437	12.15427
fatty acid elongation	0.008331	0.000879	9.481193
fatty acid metabolic process	0.173403	0.016545	10.48047
fatty acid omega-oxidation	0.001851	0.000315	5.877231
fatty acid oxidation	0.037026	0.003117	11.87951
fatty acid transport	0.006788	0.000398	17.0603

fatty-acyl-CoA biosynthetic process	0.00216	0.000166	13.02786
flavone biosynthetic process	0.015427	0.001641	9.399612
flavone metabolic process	0.015427	0.001641	9.399612
flavonoid biosynthetic process	0.079297	0.007875	10.06966
flavonoid metabolic process	0.087319	0.008803	9.91898
flavonol biosynthetic process	0.015427	0.001641	9.399612
flavonol metabolic process	0.015427	0.001641	9.399612
fructose metabolic process	0.004628	0.000448	10.33957
fructose transport	0.001543	0.000133	11.63202
galactolipid biosynthetic process	0.004628	0.000547	8.459651
galactolipid metabolic process	0.004628	0.000564	8.210837
galactose biosynthetic process	0.004937	0.000398	12.40749
galactose metabolic process	0.013576	0.001426	9.522025
GDP-mannose biosynthetic process	0.001543	0.000182	8.459651
GDP-mannose metabolic process	0.00216	0.000216	10.02143
GDP-mannose transport	0.001543	0.000116	13.29374
gene expression	1.818575	0.195179	9.317472
gene silencing	0.058007	0.007278	7.970186
gene silencing by miRNA	0.013268	0.001741	7.621742
gene silencing by RNA	0.030855	0.004078	7.565541
generation of precursor metabolites and energy	0.242209	0.022398	10.81408
gibberellic acid mediated signaling pathway	0.04042	0.004526	8.930664
gibberellin biosynthetic process	0.013576	0.001277	10.63499
gibberellin catabolic process	0.003703	0.000398	9.305616
gibberellin mediated signaling pathway	0.041037	0.004559	9.001068
gibberellin metabolic process	0.017279	0.001674	10.3191
glucan biosynthetic process	0.058932	0.006101	9.659634
glucan catabolic process	0.020673	0.002089	9.896448
glucan metabolic process	0.103672	0.010461	9.910259
gluconeogenesis	0.015736	0.001343	11.71818
glucosamine metabolic process	0.004628	0.001409	3.284335
glucose 6-phosphate metabolic process	0.001543	0.000116	13.29374
glucose catabolic process	0.083925	0.008405	9.984724
glucose homeostasis	0.004937	0.000398	12.40749
glucose import	0.001851	0.000149	12.40749
glucose mediated signaling pathway	0.009873	0.00121	8.158348
glucose metabolic process	0.108608	0.009466	11.47312
glucose transport	0.00432	0.000332	13.02786
glucoside transport	0.002468	0.000216	11.45307
glucosinolate biosynthetic process	0.015119	0.001757	8.603305
glucosinolate catabolic process	0.001543	0.000149	10.33957
glucosinolate metabolic process	0.016662	0.001907	8.739187
glutathione biosynthetic process	0.00216	0.000249	8.685241
glutathione catabolic process	0.001851	0.000116	15.95248
glutathione metabolic process	0.013576	0.00131	10.36575
mannose biosynthetic process	0.001543	0.000182	8.459651
mannose metabolic process	0.007097	0.000547	12.97146

MAPKKK cascade	0.027152	0.002487	10.91859
MAPKKK cascade involved in osmosensory signaling pathway	0.005554	0.000398	13.95842
mitochondrial ATP synthesis coupled electron transport	0.016044	0.001575	10.1872
mitochondrial DNA metabolic process	0.001543	0.000298	5.169786
mitochondrial electron transport, NADH to ubiquinone	0.008639	0.000846	10.21793
mitochondrial electron transport, succinate to ubiquinone	0.002777	0.000265	10.46882
mitochondrial electron transport, ubiquinol to cytochrome c	0.003394	0.000315	10.77492
mitochondrial fission	0.009256	0.000713	12.98458
mitochondrial genome maintenance	0.003394	0.000497	6.824118
mitochondrial membrane organization	0.006788	0.000663	10.23618
mitochondrial protein processing	0.00216	0.000182	11.84351
mitochondrial protein processing during import	0.00216	0.000182	11.84351
mitochondrial respiratory chain complex assembly	0.003703	0.000315	11.75446
mitochondrial respiratory chain complex I assembly	0.003085	0.000282	10.94778
mitochondrial transport	0.057698	0.004791	12.04256
mitochondrion distribution	0.005862	0.000497	11.78711
mitochondrion inheritance	0.004937	0.000414	11.91119
mitochondrion localization	0.008948	0.000696	12.85061
mitochondrion localization, microtubule-mediated	0.003085	0.000216	14.31633
mitochondrion morphogenesis	0.009256	0.000713	12.98458
mitochondrion organization	0.057081	0.005156	11.07099
mitochondrion transport along microtubule	0.003085	0.000216	14.31633
mRNA 3'-end processing	0.011416	0.001111	10.27784
mRNA capping	0.002777	0.000348	7.976242
mRNA catabolic process	0.038568	0.003266	11.80916
mRNA cleavage	0.006171	0.000879	7.023106
mRNA cleavage involved in gene silencing by miRNA	0.003085	0.000497	6.203744
mRNA export from nucleus	0.015119	0.00131	11.54367
mRNA metabolic process	0.183894	0.01812	10.14848
mRNA modification	0.002468	0.000348	7.089993
mRNA polyadenylation	0.005554	0.00058	9.57149
mRNA processing	0.157976	0.015335	10.30157
mRNA splice site selection	0.010182	0.000862	11.81097
mRNA stabilization	0.004628	0.000398	11.63202
mRNA transport	0.024992	0.002122	11.77742
NAD biosynthetic process	0.004011	0.000414	9.67784
NAD metabolic process	0.008331	0.000829	10.05006
NADH dehydrogenase complex (plastoquinone) assembly	0.001543	9.95E-05	15.50936
NADH dehydrogenase complex assembly	0.004628	0.000381	12.13776
NADP biosynthetic process	0.00432	0.000332	13.02786
NADP metabolic process	0.031163	0.002818	11.05726
NADPH regeneration	0.028386	0.002603	10.90594
ncRNA 3'-end processing	0.003394	0.000332	10.23618
ncRNA 5'-end processing	0.008639	0.000763	11.32858
ncRNA catabolic process	0.002468	0.000199	12.40749
ncRNA metabolic process	0.147485	0.015285	9.648773
ncRNA polyadenylation	0.001543	9.95E-05	15.50936

ncRNA polyadenylation during polyadenylation-dependent ncR	0.001543	9.95E-05	15.50936
ncRNA processing	0.105214	0.010793	9.74874
negative regulation of mRNA 3'-end processing	0.003394	0.000182	18.61123
negative regulation of mRNA processing	0.005862	0.000381	15.3745
negative regulation of RNA splicing	0.002468	0.000199	12.40749
phenylpropanoid biosynthetic process	0.133909	0.013147	10.18572
phenylpropanoid catabolic process	0.012959	0.000895	14.4754
phenylpropanoid metabolic process	0.172786	0.01696	10.18797
phloem loading	0.007405	0.00068	10.89438
phloem or xylem histogenesis	0.022832	0.002338	9.767596
phloem transport	0.003085	0.000398	7.75468
photosynthesis	0.104906	0.009085	11.54711
photosynthesis, dark reaction	0.013576	0.001243	10.91859
photosynthesis, light harvesting	0.01913	0.001376	13.90237
photosynthesis, light harvesting in photosystem I	0.007097	0.000464	15.2878
photosynthesis, light reaction	0.062018	0.005322	11.65376
photosynthetic acclimation	0.002468	0.000315	7.836308
photosynthetic electron transport chain	0.024992	0.002404	10.39662
photosynthetic electron transport in photosystem I	0.008331	0.000663	12.56258
photosystem I assembly	0.001543	8.29E-05	18.61123
photosystem I stabilization	0.003394	0.000481	7.059432
photosystem II assembly	0.007714	0.000497	15.50936
photosystem II oxygen evolving complex assembly	0.002777	0.000166	16.75011
photosystem II repair	0.003085	0.000298	10.33957
polycistronic mRNA processing	0.003085	0.000332	9.305616
polysaccharide biosynthetic process	0.105832	0.009997	10.58649
polysaccharide catabolic process	0.042888	0.004625	9.272262
polysaccharide metabolic process	0.17402	0.016976	10.25072
polysaccharide transport	0.006479	0.000448	14.4754
positive regulation of gene expression, epigenetic	0.003085	0.000497	6.203744
positive regulation of gene-specific transcription	0.005245	0.000398	13.18296
positive regulation of RNA metabolic process	0.045356	0.004725	9.599477
positive regulation of transport	0.010182	0.000812	12.53409
raffinose family oligosaccharide biosynthetic process	0.005245	0.000547	9.587604
regulation of cellular biosynthetic process	1.138846	0.125698	9.06015
regulation of cellular carbohydrate catabolic process	0.002777	0.000332	8.375054
regulation of cellular carbohydrate metabolic process	0.009565	0.000829	11.53896
regulation of cellular catabolic process	0.017279	0.001807	9.561733
regulation of glucan biosynthetic process	0.001851	0.000166	11.16674
regulation of glucose import	0.001851	0.000149	12.40749
regulation of glucose metabolic process	0.008639	0.000613	14.08417
regulation of glucose transport	0.001851	0.000149	12.40749
regulation of glycogen biosynthetic process	0.001851	0.000166	11.16674
regulation of glycogen metabolic process	0.001851	0.000166	11.16674
regulation of glycolysis	0.002777	0.000315	8.815846
regulation of growth	0.294354	0.027421	10.73465
regulation of transcription	1.044431	0.117111	8.918321

regulation of transcription by carbon catabolites	0.005245	0.000365	14.38141
regulation of transcription by glucose	0.005245	0.000348	15.06623
regulation of transcription factor activity	0.009565	0.000895	10.68423
regulation of transcription from RNA polymerase I promoter	0.001851	0.000216	8.589799
regulation of transcription from RNA polymerase II promoter	0.07436	0.007991	9.305616
regulation of transcription from RNA polymerase II promoter, g	0.001543	0.000166	9.305616
regulation of transcription from RNA polymerase II promoter, r	0.00216	0.000199	10.85655
regulation of transcription in response to stress	0.002777	0.000332	8.375054
regulation of transcription involved in G1 phase of mitotic cell c	0.004011	0.000348	11.52124
regulation of transcription regulator activity	0.009565	0.000895	10.68423
regulation of transcription termination	0.001543	0.000166	9.305616
regulation of transcription, DNA-dependent	0.670781	0.074753	8.973346
regulation of transcription, mitotic	0.00216	0.000199	10.85655
regulation of transferase activity	0.037334	0.003697	10.09847
regulation of transforming growth factor beta receptor signalin	0.003085	0.000199	15.50936
regulation of translation	0.061709	0.005305	11.63202
regulation of translation in response to stress	0.00432	0.000282	15.3269
regulation of translational elongation	0.003085	0.000332	9.305616
regulation of translational initiation	0.018204	0.001492	12.2007
regulation of translational initiation in response to stress	0.00432	0.000282	15.3269
regulation of translational termination	0.003394	0.000315	10.77492
regulation of transmembrane transport	0.008948	0.001194	7.49619
regulation of transmembrane transporter activity	0.008948	0.001194	7.49619
sucrose biosynthetic process	0.007405	0.000862	8.589799
sucrose mediated signaling	0.001543	0.000182	8.459651
sucrose metabolic process	0.011416	0.00126	9.060731
sucrose transport	0.008331	0.000746	11.16674
sugar mediated signaling pathway	0.030855	0.003432	8.990933
tissue development	0.375193	0.039706	9.449377
tissue homeostasis	0.001543	0.000298	5.169786
tissue morphogenesis	0.059241	0.005421	10.9277
tissue regeneration	0.00216	0.000166	13.02786
tissue remodeling	0.003085	0.000332	9.305616
transcription	1.143166	0.12724	8.984314
transcription factor import into nucleus	0.004937	0.000779	6.335738
UDP-galactose transport	0.003085	0.000315	9.795385
UDP-glucose transport	0.001543	0.000133	11.63202
UDP-glucuronic acid transport	0.001543	0.000133	11.63202
UDP-N-acetylglucosamine metabolic process	0.00216	0.000216	10.02143
UDP-N-acetylglucosamine transport	0.002468	0.000182	13.53544
UDP-rhamnose biosynthetic process	0.00216	0.000298	7.237701
UDP-rhamnose metabolic process	0.00216	0.000298	7.237701
UDP-xylose transport	0.001543	0.000133	11.63202
UMP biosynthetic process	0.001543	0.000133	11.63202
UMP metabolic process	0.001543	0.000133	11.63202
xyloglucan biosynthetic process	0.008948	0.001078	8.303472
