# Supplementary material for: Genome‐wide discovery of tissue‐specific miRNAs in clusterbean (Cyamopsis tetragonoloba) indicates their association with galactomannan biosynthesis
Source: Plant Biotechnol J. 2018 Mar 11;16(6):1241–57. doi: 10.1111/pbi.12866 (PMC5978871; doi:10.1111/pbi.12866)
Supplement: Supplementary file 10 — Table S9 Molecular Functions (MFs) based on singular enrichment analysis (SEA) for miRNA targeted clusterbean unigenes. The difference between the query and background ratio of a particular Gene Ontology term are shown in different colors. A Fisher's exact test with FDR corrected P value of < 0.05 was used as a parameter. [file PBI-16-1241-s004.pdf]

| Term | Qu_ratio | BG_ratio | Ratio_Q/B |
|---|---|---|---|
| carbohydrate binding | 0.003085 | 0.000216 | 14.31633 |
| carbohydrate biosynthetic process | 0.005862 | 0.000381 | 15.3745 |
| carbohydrate catabolic process | 0.003085 | 0.000216 | 14.31633 |
| carbohydrate homeostasis | 0.002468 | 0.000249 | 9.92599 |
| carbohydrate kinase activity | 0.002777 | 0.000232 | 11.96436 |
| carbohydrate mediated signaling | 0.002468 | 0.000199 | 12.40749 |
| carbohydrate metabolic process | 0.001851 | 0.000116 | 15.95248 |
| carbohydrate phosphatase activity | 0.390003 | 0.033671 | 11.58277 |
| carbohydrate phosphorylation | 0.015427 | 0.001641 | 9.399612 |
| carbohydrate transmembrane transporter activity | 0.007097 | 0.000547 | 12.97146 |
| carbohydrate transport | 0.003394 | 0.000265 | 12.79522 |
| carbon fixation | 0.001543 | 0.000133 | 11.63202 |
| carbon utilization | 0.010799 | 0.000912 | 11.84351 |
| cell wall | 0.004937 | 0.000315 | 15.67262 |
| cell wall biogenesis | 0.001543 | 0.000116 | 13.29374 |
| cell wall glycoprotein biosynthetic process | 0.001851 | 0.000232 | 7.976242 |
| cell wall macromolecule biosynthetic process | 0.007097 | 0.000613 | 11.56914 |
| cell wall macromolecule catabolic process | 0.005862 | 0.000497 | 11.78711 |
| cell wall macromolecule metabolic process | 0.003703 | 0.000298 | 12.40749 |
| cell wall modification | 0.00216 | 0.000166 | 13.02786 |
| cell wall modification during multidimensional cell growth | 0.01265 | 0.001078 | 11.73939 |
| cell wall pectin metabolic process | 0.002468 | 0.000249 | 9.92599 |
| cell wall polysaccharide biosynthetic process | 0.001851 | 0.000166 | 11.16674 |
| cell wall polysaccharide metabolic process | 0.00216 | 0.000182 | 11.84351 |
| cell wall thickening | 0.037334 | 0.003697 | 10.09847 |
| cellular carbohydrate biosynthetic process | 0.005245 | 0.000365 | 14.38141 |
| cellular carbohydrate catabolic process | 0.015736 | 0.001144 | 13.75613 |
| cellular carbohydrate metabolic process | 0.003394 | 0.000216 | 15.74796 |
| cellular cell wall organization or biogenesis | 0.001543 | 9.95E-05 | 15.50936 |
| cellular polysaccharide biosynthetic process | 0.002468 | 0.000182 | 13.53544 |
| cellular polysaccharide catabolic process | 0.003703 | 0.000315 | 11.75446 |
| cellular polysaccharide metabolic process | 0.003085 | 0.000216 | 14.31633 |
| cellular potassium ion homeostasis | 0.00216 | 0.000182 | 11.84351 |
| cellular respiration | 0.014193 | 0.001127 | 12.58995 |
| cellular response to chemical stimulus | 0.001851 | 0.000116 | 15.95248 |
| cellular response to extracellular stimulus | 0.007097 | 0.000547 | 12.97146 |
| cellular response to glucose starvation | 0.00432 | 0.000497 | 8.685241 |
| cellular response to heat | 0.003085 | 0.000497 | 6.203744 |
| cellular response to hormone stimulus | 0.020673 | 0.00131 | 15.78421 |
| cellular response to hydrogen peroxide | 0.001851 | 0.000166 | 11.16674 |
| cellular response to nitrogen levels | 0.315643 | 0.028681 | 11.00537 |
| cellular response to oxidative stress | 0.002468 | 0.000216 | 11.45307 |
| cellular response to phosphate starvation | 0.00216 | 0.000182 | 11.84351 |
| cellular response to starvation | 0.003085 | 0.000249 | 12.40749 |
| cellular response to stimulus | 0.00216 | 0.000133 | 16.28483 |
| cellular response to stress | 0.004937 | 0.000315 | 15.67262 |

| | | | |
|---|---|---|---|
| cellular response to sucrose starvation | 0.003703 | 0.000481 | 7.701199 |
| cellular response to water deprivation | 0.00432 | 0.000398 | 10.85655 |
| cellular sodium ion homeostasis | 0.017279 | 0.001674 | 10.3191 |
| cellulase activity | 0.007714 | 0.000696 | 11.07811 |
| cellulose biosynthetic process | 0.008639 | 0.000729 | 11.84351 |
| cellulose catabolic process | 0.510336 | 0.048111 | 10.6075 |
| cellulose metabolic process | 0.006788 | 0.000564 | 12.04256 |
| cellulose microfibril organization | 0.029929 | 0.002735 | 10.94115 |
| cellulose synthase (UDP-forming) activity | 0.061709 | 0.005305 | 11.63202 |
| cellulose synthase activity | 0.00216 | 0.000182 | 11.84351 |
| cellulose synthase complex | 0.008948 | 0.001194 | 7.49619 |
| channel activity | 0.005245 | 0.000398 | 13.18296 |
| chaperone activator activity | 0.007405 | 0.000862 | 8.589799 |
| chaperone binding | 0.052144 | 0.004675 | 11.15354 |
| chaperone mediated protein folding requiring cofactor | 0.024992 | 0.002404 | 10.39662 |
| chaperone-mediated protein complex assembly | 0.00216 | 0.000282 | 7.663448 |
| chlorophyll binding | 0.174637 | 0.015667 | 11.14704 |
| chlorophyll metabolic process | 0.008331 | 0.00068 | 12.25618 |
| chloroplast ribulose bisphosphate carboxylase complex | 0.006171 | 0.00063 | 9.795385 |
| chloroplastic endopeptidase Clp complex | 0.00216 | 0.000216 | 10.02143 |
| chromatin assembly | 0.001851 | 0.000149 | 12.40749 |
| chromatin binding | 0.014502 | 0.001111 | 13.05564 |
| chromatin disassembly | 0.001543 | 0.000166 | 9.305616 |
| chromatin remodeling | 0.005245 | 0.000431 | 12.16888 |
| chromatin remodeling at centromere | 0.00216 | 0.000282 | 7.663448 |
| chromatin remodeling complex | 0.005554 | 0.000448 | 12.40749 |
| chromatin silencing | 0.004937 | 0.000398 | 12.40749 |
| chromatin silencing by small RNA | 0.048442 | 0.004675 | 10.36157 |
| defense response | 0.001851 | 0.000116 | 15.95248 |
| defense response signaling pathway, resistance gene-dependent | 0.049985 | 0.00441 | 11.33466 |
| defense response signaling pathway, resistance gene-independent | 0.149337 | 0.013346 | 11.18986 |
| defense response to bacterium | 0.003085 | 0.000216 | 14.31633 |
| defense response to bacterium, incompatible interaction | 0.022832 | 0.00184 | 12.40749 |
| defense response to fungus | 0.008639 | 0.000613 | 14.08417 |
| defense response to fungus, incompatible interaction | 0.061401 | 0.005222 | 11.75757 |
| defense response to insect | 0.007714 | 0.000746 | 10.33957 |
| defense response to oomycetes | 0.012033 | 0.00116 | 10.36911 |
| defense response to virus | 0.004628 | 0.000381 | 12.13776 |
| defense response, incompatible interaction | 0.003703 | 0.000265 | 13.95842 |
| fatty acid (omega-1)-hydroxylase activity | 0.19963 | 0.01938 | 10.30066 |
| fatty acid alpha-oxidation | 0.018204 | 0.002122 | 8.578614 |
| fatty acid beta-oxidation | 0.010182 | 0.000862 | 11.81097 |
| fatty acid binding | 0.001851 | 0.000166 | 11.16674 |
| fatty acid biosynthetic process | 0.005554 | 0.000398 | 13.95842 |
| fatty acid catabolic process | 0.037026 | 0.003117 | 11.87951 |
| fatty acid elongase activity | 0.013576 | 0.00131 | 10.36575 |
| fatty acid elongation | 0.013576 | 0.001243 | 10.91859 |

| | | | |
|---|---|---|---|
| fatty acid metabolic process | 0.00216 | 0.000149 | 14.4754 |
| fatty acid omega-oxidation | 0.018513 | 0.001956 | 9.463338 |
| fatty acid oxidation | 0.057698 | 0.004791 | 12.04256 |
| fatty acid synthase complex | 0.459118 | 0.054228 | 8.466375 |
| fatty-acid ligase activity | 0.001851 | 0.000166 | 11.16674 |
| fatty-acid synthase activity | 0.008022 | 0.000696 | 11.52124 |
| fatty-acyl-CoA biosynthetic process | 0.002777 | 0.000348 | 7.976242 |
| fatty-acyl-CoA synthase activity | 0.013576 | 0.001277 | 10.63499 |
| flavin-containing monooxygenase activity | 0.173403 | 0.016545 | 10.48047 |
| flavone biosynthetic process | 0.001543 | 0.000133 | 11.63202 |
| flavone metabolic process | 0.001543 | 0.000133 | 11.63202 |
| flavonoid biosynthetic process | 0.001543 | 0.000116 | 13.29374 |
| flavonoid metabolic process | 0.003085 | 0.000232 | 13.29374 |
| flavonol 3-O-glucosyltransferase activity | 0.001543 | 0.000149 | 10.33957 |
| flavonol biosynthetic process | 0.001543 | 0.000133 | 11.63202 |
| flavonol metabolic process | 0.001543 | 0.000133 | 11.63202 |
| flavonol synthase activity | 0.013576 | 0.001426 | 9.522025 |
| fructokinase activity | 0.009565 | 0.000895 | 10.68423 |
| fructose 1,6-bisphosphate 1-phosphatase activity | 0.015119 | 0.001757 | 8.603305 |
| fructose metabolic process | 0.037951 | 0.003763 | 10.0845 |
| fructose transmembrane transporter activity | 0.002468 | 0.000348 | 7.089993 |
| fructose transport | 0.003394 | 0.000481 | 7.059432 |
| fructose-2,6-bisphosphate 2-phosphatase activity | 0.006479 | 0.000663 | 9.770896 |
| fructose-bisphosphate aldolase activity | 0.00216 | 0.000216 | 10.02143 |
| fucosyltransferase activity | 0.297748 | 0.029228 | 10.18709 |
| galactinol-sucrose galactosyltransferase activity | 0.001543 | 0.000199 | 7.75468 |
| galactokinase activity | 0.030855 | 0.003432 | 8.990933 |
| galactolipase activity | 0.005245 | 0.00068 | 7.716852 |
| galactolipid biosynthetic process | 0.011416 | 0.001111 | 10.27784 |
| galactolipid galactosyltransferase activity | 0.00432 | 0.000464 | 9.305616 |
| galactolipid metabolic process | 0.052144 | 0.005056 | 10.31245 |
| galactose biosynthetic process | 0.087319 | 0.008803 | 9.91898 |
| galactose metabolic process | 0.009565 | 0.000829 | 11.53896 |
| galactose transmembrane transporter activity | 0.006788 | 0.000663 | 10.23618 |
| galactosidase activity | 0.005862 | 0.000497 | 11.78711 |
| galactoside 2-alpha-L-fucosyltransferase activity | 0.042888 | 0.004625 | 9.272262 |
| galactosylgalactosylxylosylprotein 3-beta-glucuronosyltransferase activity | 0.030238 | 0.003299 | 9.16533 |
| galactosyltransferase activity | 0.00216 | 0.000182 | 11.84351 |
| galacturan 1,4-alpha-galacturonidase activity | 0.003703 | 0.000398 | 9.305616 |
| gated channel activity | 0.004937 | 0.000398 | 12.40749 |
| GDP binding | 0.007097 | 0.000796 | 8.917882 |
| GDP-dissociation inhibitor activity | 0.003085 | 0.000298 | 10.33957 |
| GDP-fucose transmembrane transporter activity | 0.006171 | 0.000879 | 7.023106 |
| GDP-mannose 3,5-epimerase activity | 0.002777 | 0.000315 | 8.815846 |
| GDP-mannose biosynthetic process | 0.010182 | 0.00121 | 8.413296 |
| GDP-mannose metabolic process | 0.00216 | 0.000249 | 8.685241 |
| GDP-mannose transmembrane transporter activity | 1.138846 | 0.125698 | 9.06015 |

| | | | |
|---|---|---|---|
| GDP-mannose transport | 0.132984 | 0.021453 | 6.198949 |
| gene expression | 0.001543 | 8.29E-05 | 18.61123 |
| gene silencing | 0.04875 | 0.003714 | 13.12756 |
| gene silencing by miRNA | 0.001543 | 0.000133 | 11.63202 |
| gene silencing by RNA | 0.001851 | 0.000149 | 12.40749 |
| generation of precursor metabolites and energy | 0.001543 | 9.95E-05 | 15.50936 |
| gibberellic acid mediated signaling pathway | 0.001851 | 0.000149 | 12.40749 |
| gibberellin 20-oxidase activity | 0.001543 | 0.000166 | 9.305616 |
| gibberellin 2-beta-dioxygenase activity | 0.004628 | 0.000448 | 10.33957 |
| gibberellin 3-beta-dioxygenase activity | 0.131441 | 0.013578 | 9.680567 |
| gibberellin biosynthetic process | 0.008639 | 0.000763 | 11.32858 |
| gibberellin catabolic process | 0.020056 | 0.002023 | 9.91582 |
| gibberellin mediated signaling pathway | 0.001851 | 0.000149 | 12.40749 |
| gibberellin metabolic process | 0.001543 | 0.000133 | 11.63202 |
| glucan 1,3-beta-glucosidase activity | 0.031163 | 0.003548 | 8.783805 |
| glucan biosynthetic process | 0.002777 | 0.000216 | 12.8847 |
| glucan catabolic process | 0.038568 | 0.003266 | 11.80916 |
| glucan endo-1,3-beta-D-glucosidase activity | 0.003394 | 0.000315 | 10.77492 |
| glucan metabolic process | 0.02345 | 0.001708 | 13.73256 |
| glucocorticoid receptor binding | 0.003085 | 0.000348 | 8.862491 |
| glucokinase activity | 0.005554 | 0.000547 | 10.15158 |
| gluconeogenesis | 0.026226 | 0.002288 | 11.46344 |
| glucosamine metabolic process | 0.009565 | 0.000829 | 11.53896 |
| glucose 6-phosphate metabolic process | 0.007097 | 0.001177 | 6.02899 |
| glucose binding | 0.002777 | 0.000398 | 6.979212 |
| glucose catabolic process | 0.001543 | 0.000116 | 13.29374 |
| glucose homeostasis | 0.103672 | 0.010461 | 9.910259 |
| glucose import | 0.00216 | 0.000282 | 7.663448 |
| glucose mediated signaling pathway | 0.065103 | 0.005819 | 11.18795 |
| glucose metabolic process | 0.002468 | 0.000182 | 13.53544 |
| glucose transmembrane transporter activity | 0.108608 | 0.009466 | 11.47312 |
| glucose transport | 0.025918 | 0.002686 | 9.650268 |
| glucose-1-phosphate adenylyltransferase activity | 0.021598 | 0.002271 | 9.509388 |
| glucose-1-phosphate adenylyltransferase complex | 0.004628 | 0.000564 | 8.210837 |
| glucose-6-phosphate dehydrogenase activity | 0.003703 | 0.000398 | 9.305616 |
| glucose-6-phosphate isomerase activity | 1.143166 | 0.12724 | 8.984314 |
| glucose-6-phosphate transmembrane transporter activity | 1.818575 | 0.195179 | 9.317472 |
| glucosidase activity | 0.104906 | 0.009085 | 11.54711 |
| glucoside transport | 0.00216 | 0.000249 | 8.685241 |
| glucosinolate biosynthetic process | 0.062018 | 0.005322 | 11.65376 |
| glucosinolate catabolic process | 0.00216 | 0.000282 | 7.663448 |
| glucosinolate metabolic process | 0.024992 | 0.002122 | 11.77742 |
| glucosyltransferase activity | 0.034557 | 0.002619 | 13.19277 |
| glutathione biosynthetic process | 0.033323 | 0.00368 | 9.054112 |
| glutathione catabolic process | 0.001851 | 0.000315 | 5.877231 |
| glutathione disulfide oxidoreductase activity | 0.139155 | 0.013893 | 10.01631 |
| glutathione gamma-glutamylcysteinyltransferase activity | 0.04042 | 0.004526 | 8.930664 |

| | | | |
|---|---|---|---|
| glutathione metabolic process | 0.253625 | 0.022182 | 11.43381 |
| glutathione peroxidase activity | 0.825363 | 0.078516 | 10.51204 |
| glutathione S-conjugate-exporting ATPase activity | 0.120333 | 0.011058 | 10.88213 |
| glutathione synthase activity | 0.001851 | 0.000282 | 6.56867 |
| glutathione transferase activity | 0.00216 | 0.000182 | 11.84351 |
| mannose binding | 0.00216 | 0.000249 | 8.685241 |
| mannose biosynthetic process | 0.008331 | 0.000995 | 8.375054 |
| mannose metabolic process | 0.009873 | 0.000962 | 10.26827 |
| mannose-1-phosphate guanylyltransferase (GDP) activity | 0.008948 | 0.001194 | 7.49619 |
| mannose-phosphate guanylyltransferase activity | 0.009873 | 0.00121 | 8.158348 |
| mannosidase activity | 0.031163 | 0.002818 | 11.05726 |
| mannosyl-glycoprotein endo-beta-N-acetylglucosaminidase activity | 0.021907 | 0.002686 | 8.156774 |
| mannosyl-oligosaccharide 1,2-alpha-mannosidase activity | 0.003085 | 0.000315 | 9.795385 |
| mannosyl-oligosaccharide mannosidase activity | 0.062018 | 0.0062 | 10.00229 |
| mannosyltransferase activity | 0.220611 | 0.019795 | 11.14492 |
| mannosyltransferase complex | 0.094107 | 0.011588 | 8.12078 |
| MAP kinase activity | 0.006788 | 0.000613 | 11.06614 |
| MAP kinase kinase activity | 0.079297 | 0.007875 | 10.06966 |
| MAP kinase kinase kinase activity | 0.005554 | 0.000514 | 10.80652 |
| MAPKKK cascade | 0.004937 | 0.000414 | 11.91119 |
| MAPKKK cascade involved in osmosensory signaling pathway | 0.020673 | 0.002089 | 9.896448 |
| mitochondrial ATP synthesis coupled electron transport | 0.039494 | 0.003399 | 11.62067 |
| mitochondrial DNA metabolic process | 0.017279 | 0.001857 | 9.305616 |
| mitochondrial electron transport, NADH to ubiquinone | 0.025918 | 0.002387 | 10.85655 |
| mitochondrial electron transport, succinate to ubiquinone | 0.001543 | 0.000166 | 9.305616 |
| mitochondrial electron transport, ubiquinol to cytochrome c | 0.269978 | 0.028532 | 9.462421 |
| mitochondrial envelope | 0.005245 | 0.000348 | 15.06623 |
| mitochondrial fission | 0.105832 | 0.009997 | 10.58649 |
| mitochondrial genome maintenance | 0.003394 | 0.000332 | 10.23618 |
| mitochondrial inner membrane | 0.014193 | 0.000995 | 14.26861 |
| mitochondrial inner membrane presequence translocase complex | 0.001851 | 0.000166 | 11.16674 |
| mitochondrial intermembrane space | 0.001851 | 0.000166 | 11.16674 |
| mitochondrial intermembrane space protein transporter complex | 0.013576 | 0.001459 | 9.305616 |
| mitochondrial lumen | 0.009256 | 0.000713 | 12.98458 |
| mitochondrial matrix | 0.009256 | 0.000713 | 12.98458 |
| mitochondrial membrane | 0.00216 | 0.000149 | 14.4754 |
| mitochondrial membrane organization | 0.034557 | 0.003316 | 10.42229 |
| mitochondrial membrane part | 0.002777 | 0.000216 | 12.8847 |
| mitochondrial nucleoid | 0.002777 | 0.000348 | 7.976242 |
| mitochondrial outer membrane | 0.010799 | 0.000912 | 11.84351 |
| mitochondrial outer membrane translocase complex | 0.012033 | 0.001243 | 9.67784 |
| mitochondrial oxoglutarate dehydrogenase complex | 0.004937 | 0.000779 | 6.335738 |
| mitochondrial part | 0.001543 | 9.95E-05 | 15.50936 |
| mitochondrial processing peptidase activity | 0.004937 | 0.000564 | 8.758226 |
| mitochondrial protein processing | 0.002777 | 0.000332 | 8.375054 |
| mitochondrial protein processing during import | 0.002777 | 0.000332 | 8.375054 |
| mitochondrial proton-transporting ATP synthase complex | 0.009565 | 0.000895 | 10.68423 |

| | | | |
|---|---|---|---|
| mitochondrial proton-transporting ATP synthase complex, catalytic core I | 0.022832 | 0.002338 | 9.767596 |
| mitochondrial pyruvate dehydrogenase complex | 0.004011 | 0.000414 | 9.67784 |
| mitochondrial respiratory chain | 0.051527 | 0.004294 | 12.00029 |
| mitochondrial respiratory chain complex assembly | 0.021598 | 0.002288 | 9.44048 |
| mitochondrial respiratory chain complex I | 0.006788 | 0.000647 | 10.49864 |
| mitochondrial respiratory chain complex I assembly | 0.001851 | 0.000199 | 9.305616 |
| mitochondrial respiratory chain complex II | 0.034866 | 0.003863 | 9.026048 |
| mitochondrial respiratory chain complex III | 0.002777 | 0.000332 | 8.375054 |
| mitochondrial respiratory chain complex IV | 0.003394 | 0.000497 | 6.824118 |
| mitochondrial ribosome | 0.028386 | 0.002603 | 10.90594 |
| mitochondrial small ribosomal subunit | 0.008022 | 0.000812 | 9.875347 |
| mitochondrial transport | 0.029003 | 0.002321 | 12.49611 |
| mitochondrial tricarboxylic acid cycle enzyme complex | 0.157976 | 0.017905 | 8.823102 |
| mitochondrion | 0.00216 | 0.000133 | 16.28483 |
| mitochondrion distribution | 0.044122 | 0.00431 | 10.23618 |
| mitochondrion inheritance | 0.00216 | 0.000216 | 10.02143 |
| mitochondrion localization | 0.099352 | 0.009466 | 10.4953 |
| mitochondrion localization, microtubule-mediated | 0.061709 | 0.007112 | 8.676565 |
| mitochondrion morphogenesis | 0.015427 | 0.001459 | 10.57456 |
| mitochondrion organization | 0.026226 | 0.002089 | 12.5552 |
| mitochondrion transport along microtubule | 0.006171 | 0.000713 | 8.656387 |
| mRNA 3'-end processing | 0.010182 | 0.000912 | 11.16674 |
| mRNA 3'-UTR binding | 0.157976 | 0.015335 | 10.30157 |
| mRNA 5'-UTR binding | 0.101512 | 0.011754 | 8.636241 |
| mRNA binding | 0.001851 | 0.000149 | 12.40749 |
| mRNA cap binding complex | 0.002468 | 0.000365 | 6.76772 |
| mRNA capping | 0.580376 | 0.05965 | 9.729774 |
| mRNA catabolic process | 0.02962 | 0.002437 | 12.15427 |
| mRNA cleavage | 0.01481 | 0.001359 | 10.89438 |
| mRNA cleavage and polyadenylation specificity factor complex | 0.147485 | 0.015285 | 9.648773 |
| mRNA cleavage factor complex | 0.008639 | 0.000846 | 10.21793 |
| mRNA cleavage involved in gene silencing by miRNA | 0.013885 | 0.001359 | 10.21348 |
| mRNA export from nucleus | 0.099044 | 0.008704 | 11.37944 |
| mRNA metabolic process | 0.00432 | 0.000282 | 15.3269 |
| mRNA methyltransferase activity | 0.003085 | 0.000497 | 6.203744 |
| mRNA modification | 0.125887 | 0.012948 | 9.722641 |
| mRNA polyadenylation | 0.142549 | 0.01381 | 10.3222 |
| mRNA processing | 0.012959 | 0.000895 | 14.4754 |
| mRNA splice site selection | 0.007405 | 0.00068 | 10.89438 |
| mRNA stabilization | 0.021907 | 0.002222 | 9.861175 |
| mRNA transport | 0.07436 | 0.006283 | 11.83458 |
| NAD biosynthetic process | 0.00216 | 0.000216 | 10.02143 |
| NAD metabolic process | 0.00216 | 0.000199 | 10.85655 |
| NAD or NADH binding | 0.001851 | 0.000149 | 12.40749 |
| NAD(P)H oxidase activity | 0.083925 | 0.008405 | 9.984724 |
| NAD+ ADP-ribosyltransferase activity | 0.023758 | 0.002321 | 10.23618 |
| NAD+ kinase activity | 0.016662 | 0.001907 | 8.739187 |

| | | | |
|---|---|---|---|
| NADH dehydrogenase (quinone) activity | 0.003703 | 0.000332 | 11.16674 |
| NADH dehydrogenase (ubiquinone) activity | 0.003703 | 0.000332 | 11.16674 |
| NADH dehydrogenase activity | 0.017587 | 0.001492 | 11.78711 |
| NADH dehydrogenase complex | 0.018513 | 0.001608 | 11.5121 |
| NADH dehydrogenase complex (plastoquinone) assembly | 0.001543 | 0.000298 | 5.169786 |
| NADH dehydrogenase complex assembly | 0.002777 | 0.000282 | 9.853005 |
| NADH kinase activity | 0.01265 | 0.001558 | 8.117665 |
| NADP biosynthetic process | 0.045356 | 0.004725 | 9.599477 |
| NADP metabolic process | 0.003394 | 0.000282 | 12.04256 |
| NADP or NADPH binding | 0.00216 | 0.000182 | 11.84351 |
| NADPH dehydrogenase activity | 0.004628 | 0.000547 | 8.459651 |
| NADPH regeneration | 0.026535 | 0.002222 | 11.94452 |
| NADPH:quinone reductase activity | 0.019747 | 0.00189 | 10.44841 |
| NADPH-hemoprotein reductase activity | 0.015427 | 0.001641 | 9.399612 |
| ncRNA 3'-end processing | 0.116322 | 0.012119 | 9.598405 |
| ncRNA 5'-end processing | 0.008331 | 0.000779 | 10.69156 |
| ncRNA catabolic process | 0.004937 | 0.00058 | 8.507991 |
| ncRNA metabolic process | 0.006479 | 0.000448 | 14.4754 |
| ncRNA polyadenylation | 0.001543 | 0.000398 | 3.87734 |
| ncRNA polyadenylation during polyadenylation-dependent ncRNA catabo | 0.004628 | 0.001409 | 3.284335 |
| ncRNA processing | 0.01913 | 0.001376 | 13.90237 |
| negative regulation of mRNA 3'-end processing | 0.077137 | 0.009218 | 8.368359 |
| negative regulation of mRNA processing | 0.05091 | 0.005172 | 9.842478 |
| negative regulation of RNA splicing | 0.623264 | 0.07341 | 8.490218 |
| phenylpropanoid biosynthetic process | 0.004011 | 0.000282 | 14.23212 |
| phenylpropanoid catabolic process | 0.058932 | 0.005388 | 10.93768 |
| phenylpropanoid metabolic process | 0.003394 | 0.000232 | 14.62311 |
| phloem loading | 0.002777 | 0.000265 | 10.46882 |
| phloem or xylem histogenesis | 0.00432 | 0.000365 | 11.84351 |
| phloem transport | 0.146868 | 0.014904 | 9.854222 |
| phosphofructokinase activity | 0.07004 | 0.006316 | 11.08858 |
| phosphoglucomutase activity | 0.147177 | 0.016065 | 9.161566 |
| phosphogluconate dehydrogenase (decarboxylating) activity | 0.008331 | 0.000829 | 10.05006 |
| phosphoglycerate dehydrogenase activity | 0.016662 | 0.002072 | 8.040052 |
| phosphoglycerate kinase activity | 0.001543 | 0.000298 | 5.169786 |
| phosphoglycerate mutase activity | 0.007097 | 0.000713 | 9.954845 |
| photosynthesis | 0.01481 | 0.001111 | 13.33342 |
| photosynthesis, dark reaction | 0.018821 | 0.001674 | 11.24045 |
| photosynthesis, light harvesting | 0.004011 | 0.000348 | 11.52124 |
| photosynthesis, light harvesting in photosystem I | 0.183894 | 0.01812 | 10.14848 |
| photosynthesis, light reaction | 0.004628 | 0.000365 | 12.68948 |
| photosynthetic acclimation | 0.059241 | 0.006283 | 9.428381 |
| photosynthetic electron transport chain | 0.025918 | 0.002188 | 11.84351 |
| photosynthetic electron transport in photosystem I | 0.00432 | 0.000414 | 10.42229 |
| photosynthetic membrane | 0.016662 | 0.001078 | 15.46164 |
| photosystem | 0.004937 | 0.000398 | 12.40749 |
| photosystem I | 0.001543 | 0.000133 | 11.63202 |

| | | | |
|---|---|---|---|
| photosystem I antenna complex | 0.011108 | 0.001194 | 9.305616 |
| photosystem I assembly | 0.005245 | 0.002172 | 2.415198 |
| photosystem I reaction center | 0.062326 | 0.006416 | 9.714389 |
| photosystem I stabilization | 0.133909 | 0.013147 | 10.18572 |
| photosystem II | 0.002777 | 0.000232 | 11.96436 |
| photosystem II antenna complex | 0.008948 | 0.001078 | 8.303472 |
| photosystem II assembly | 0.172786 | 0.01696 | 10.18797 |
| photosystem II oxygen evolving complex assembly | 0.058007 | 0.007278 | 7.970186 |
| photosystem II repair | 0.011108 | 0.001194 | 9.305616 |
| polycistronic mRNA processing | 0.010491 | 0.001094 | 9.587604 |
| polygalacturonase activity | 0.002777 | 0.000232 | 11.96436 |
| polygalacturonate 4-alpha-galacturonosyltransferase activity | 0.015119 | 0.00131 | 11.54367 |
| polysaccharide binding | 0.057081 | 0.005156 | 11.07099 |
| polysaccharide biosynthetic process | 0.003394 | 0.000249 | 13.64824 |
| polysaccharide catabolic process | 0.003085 | 0.000249 | 12.40749 |
| polysaccharide metabolic process | 0.005862 | 0.000398 | 14.73389 |
| polysaccharide transport | 0.356989 | 0.035445 | 10.07165 |
| positive regulation of gene expression, epigenetic | 0.016044 | 0.001575 | 10.1872 |
| positive regulation of gene-specific transcription | 0.008331 | 0.000846 | 9.853005 |
| positive regulation of RNA metabolic process | 0.003703 | 0.000298 | 12.40749 |
| positive regulation of transport | 0.003394 | 0.000315 | 10.77492 |
| raffinose family oligosaccharide biosynthetic process | 0.17402 | 0.016976 | 10.25072 |
| regulation of cellular biosynthetic process | 0.002777 | 0.000166 | 16.75011 |
| regulation of cellular carbohydrate catabolic process | 0.005245 | 0.000547 | 9.587604 |
| regulation of cellular carbohydrate metabolic process | 0.163838 | 0.015136 | 10.82428 |
| regulation of cellular catabolic process | 0.015736 | 0.001343 | 11.71818 |
| regulation of glucan biosynthetic process | 0.004937 | 0.00063 | 7.836308 |
| regulation of gluconeogenesis | 0.001543 | 0.000166 | 9.305616 |
| regulation of glucose import | 0.013268 | 0.001741 | 7.621742 |
| regulation of glucose metabolic process | 0.003085 | 0.000298 | 10.33957 |
| regulation of glucose transport | 0.030855 | 0.004078 | 7.565541 |
| regulation of glycogen biosynthetic process | 0.004937 | 0.00063 | 7.836308 |
| regulation of glycogen metabolic process | 0.002468 | 0.000315 | 7.836308 |
| regulation of glycolysis | 0.012959 | 0.001376 | 9.417731 |
| regulation of growth | 0.001543 | 9.95E-05 | 15.50936 |
| regulation of transcription | 0.004937 | 0.000298 | 16.54332 |
| regulation of transcription by carbon catabolites | 0.031163 | 0.003183 | 9.790283 |
| regulation of transcription by glucose | 0.007714 | 0.000796 | 9.69335 |
| regulation of transcription factor activity | 0.059241 | 0.005421 | 10.9277 |
| regulation of transcription in response to stress | 0.005554 | 0.00058 | 9.57149 |
| regulation of transcription involved in G1 phase of mitotic cell cycle | 0.105214 | 0.010793 | 9.74874 |
| regulation of transcription regulator activity | 0.027152 | 0.002487 | 10.91859 |
| regulation of transferase activity | 0.004937 | 0.000398 | 12.40749 |
| regulation of translation | 0.011108 | 0.000879 | 12.64159 |
| regulation of translational elongation | 0.017279 | 0.001807 | 9.561733 |
| regulation of translational initiation | 0.0722 | 0.00625 | 11.5518 |
| regulation of translational initiation in response to stress | 0.001543 | 0.000166 | 9.305616 |

| | | | |
|---|---|---|---|
| regulation of translational termination | 0.015427 | 0.001641 | 9.399612 |
| regulation of transmembrane transport | 0.003703 | 0.000332 | 11.16674 |
| regulation of transmembrane transporter activity | 0.001851 | 0.000166 | 11.16674 |
| sucrose biosynthetic process | 0.03178 | 0.002918 | 10.8918 |
| sucrose mediated signaling | 0.117865 | 0.014258 | 8.266849 |
| sucrose metabolic process | 0.195002 | 0.017308 | 11.26657 |
| sucrose synthase activity | 0.004011 | 0.000414 | 9.67784 |
| sucrose transmembrane transporter activity | 0.242209 | 0.022398 | 10.81408 |
| sucrose transport | 0.009565 | 0.000895 | 10.68423 |
| sucrose:hydrogen symporter activity | 0.004628 | 0.000431 | 10.73725 |
| sucrose-phosphate synthase activity | 0.058932 | 0.006101 | 9.659634 |
| sugar binding | 0.002468 | 0.000182 | 13.53544 |
| sugar mediated signaling pathway | 0.002468 | 0.000199 | 12.40749 |
| sugar transmembrane transporter activity | 0.008948 | 0.000696 | 12.85061 |
| sugar:hydrogen symporter activity | 0.010182 | 0.000812 | 12.53409 |
| tissue development | 0.001851 | 0.000116 | 15.95248 |
| tissue homeostasis | 0.008948 | 0.000962 | 9.305616 |
| tissue morphogenesis | 0.003394 | 0.000265 | 12.79522 |
| tissue regeneration | 0.003085 | 0.000398 | 7.75468 |
| tissue remodeling | 0.063869 | 0.006681 | 9.559615 |
| transcription | 0.003394 | 0.000199 | 17.0603 |
| transcription activator activity | 0.00216 | 0.000149 | 14.4754 |
| transcription antitermination | 0.001543 | 0.000199 | 7.75468 |
| transcription coactivator activity | 0.018204 | 0.001492 | 12.2007 |
| transcription cofactor activity | 0.00432 | 0.000332 | 13.02786 |
| transcription corepressor activity | 0.07436 | 0.006283 | 11.83458 |
| transcription elongation factor complex | 0.11879 | 0.011257 | 10.55276 |
| transcription elongation regulator activity | 0.052144 | 0.004741 | 10.99755 |
| transcription factor activity | 0.006479 | 0.000398 | 16.28483 |
| transcription factor binding | 0.005554 | 0.000398 | 13.95842 |
| transcription factor complex | 0.008331 | 0.000647 | 12.8847 |
| transcription factor import into nucleus | 0.294354 | 0.027421 | 10.73465 |
| transcription factor TFIIA complex | 0.064178 | 0.007278 | 8.818078 |
| transcription factor TFIID complex | 0.008331 | 0.000746 | 11.16674 |
| transcription factor TFIIE complex | 0.004628 | 0.000514 | 9.005434 |
| transcription factor TFIIF complex | 0.041037 | 0.004559 | 9.001068 |
| transcription factor TFIIIB complex | 0.005862 | 0.000713 | 8.223567 |
| transcription from plastid promoter | 0.008331 | 0.000829 | 10.05006 |
| transcription from RNA polymerase I promoter | 0.001851 | 0.000166 | 11.16674 |
| transcription from RNA polymerase II promoter | 0.004937 | 0.000348 | 14.17999 |
| transcription from RNA polymerase III promoter | 0.017896 | 0.001592 | 11.24429 |
| transcription initiation | 0.008331 | 0.000663 | 12.56258 |
| transcription initiation factor activity | 0.003085 | 0.000282 | 10.94778 |
| transcription initiation from RNA polymerase II promoter | 0.004937 | 0.000398 | 12.40749 |
| transcription initiation from RNA polymerase III promoter | 0.008331 | 0.000912 | 9.136423 |
| transcription regulator activity | 0.00216 | 0.000133 | 16.28483 |
| transcription repressor activity | 0.004628 | 0.000332 | 13.95842 |

| | | | |
|---|---|---|---|
| transferase activity | 0.003394 | 0.000182 | 18.61123 |
| transferase activity, transferring glycosyl groups | 0.007714 | 0.000497 | 15.50936 |
| UDP-4-keto-6-deoxy-glucose-3,5-epimerase activity | 0.003085 | 0.000332 | 9.305616 |
| UDP-4-keto-rhamnose-4-keto-reductase activity | 0.003085 | 0.000332 | 9.305616 |
| UDP-galactose transmembrane transporter activity | 0.05955 | 0.006167 | 9.655827 |
| UDP-galactose transport | 0.008331 | 0.000879 | 9.481193 |
| UDP-galactose:N-glycan beta-1,3-galactosyltransferase activity | 0.001851 | 0.000232 | 7.976242 |
| UDP-galactosyltransferase activity | 0.003394 | 0.000315 | 10.77492 |
| UDP-glucose 4,6-dehydratase activity | 0.003085 | 0.000332 | 9.305616 |
| UDP-glucose 4-epimerase activity | 0.011725 | 0.001144 | 10.24966 |
| UDP-glucose 6-dehydrogenase activity | 0.032089 | 0.00368 | 8.718775 |
| UDP-glucose transmembrane transporter activity | 0.001543 | 0.000182 | 8.459651 |
| UDP-glucose transport | 0.001851 | 0.000249 | 7.444492 |
| UDP-glucosyltransferase activity | 0.00432 | 0.000332 | 13.02786 |
| UDP-glucuronate 4-epimerase activity | 0.074668 | 0.007626 | 9.791126 |
| UDP-glucuronate decarboxylase activity | 0.11046 | 0.010743 | 10.28213 |
| UDP-glucuronic acid transmembrane transporter activity | 0.011725 | 0.001293 | 9.06701 |
| UDP-glucuronic acid transport | 0.001851 | 0.000249 | 7.444492 |
| UDP-glycosyltransferase activity | 0.013268 | 0.000928 | 14.29077 |
| UDP-L-rhamnose synthase activity | 0.003085 | 0.000332 | 9.305616 |
| UDP-N-acetylglucosamine transmembrane transporter activity | 0.001543 | 0.000182 | 8.459651 |
| UDP-N-acetylglucosamine transport | 0.001543 | 0.000182 | 8.459651 |
| UDP-rhamnose biosynthetic process | 0.015427 | 0.001641 | 9.399612 |
| UDP-rhamnose metabolic process | 0.120642 | 0.012848 | 9.389666 |
| UDP-xylose transmembrane transporter activity | 0.011416 | 0.00126 | 9.060731 |
| UDP-xylose transport | 0.00216 | 0.000298 | 7.237701 |
| UDP-xylosyltransferase activity | 0.002468 | 0.000249 | 9.92599 |
| xyloglucan biosynthetic process | 0.005554 | 0.000497 | 11.16674 |
| xyloglucan:xyloglucosyl transferase activity | 0.004628 | 0.000398 | 11.63202 |
| xylosyltransferase activity | 0.005554 | 0.000531 | 10.46882 |
