# Supplementary material for: Genome‐wide discovery of tissue‐specific miRNAs in clusterbean (Cyamopsis tetragonoloba) indicates their association with galactomannan biosynthesis
Source: Plant Biotechnol J. 2018 Mar 11;16(6):1241–57. doi: 10.1111/pbi.12866 (PMC5978871; doi:10.1111/pbi.12866)
Supplement: Supplementary file 11 — Table S10 Cellular Components (CCs) based on singular enrichment analysis (SEA) for miRNA targeted clusterbean unigenes. The difference between the query and background ratio of a particular Gene Ontology term are shown in different colors. [file PBI-16-1241-s003.pdf]

Term	**Qu_ratio**	**BG_ratio**	**Ratio_Q/B**
photosystem I antenna complex	0.004937	0.000298	16.54332
cellulose synthase complex	0.00216	0.000133	16.28483
mitochondrial respiratory chain complex IV	0.00216	0.000133	16.28483
mitochondrial oxoglutarate dehydrogenase complex	0.001851	0.000116	15.95248
photosystem I reaction center	0.004937	0.000348	14.17999
mitochondrial intermembrane space	0.015736	0.001144	13.75613
photosystem I	0.02345	0.001708	13.73256
photosystem II antenna complex	0.002468	0.000182	13.53544
mitochondrial inner membrane presequence translocase complex	0.01481	0.001111	13.33342
photosystem II	0.034557	0.002619	13.19277
chloroplast photosystem II	0.010491	0.000796	13.18296
photosystem	0.04875	0.003714	13.12756
chloroplast photosystem I	0.007097	0.000547	12.97146
chloroplast inner membrane	0.042888	0.003316	12.93481
mitochondrial intermembrane space protein transporter complex	0.003394	0.000265	12.79522
mitochondrial ribosome	0.011108	0.000879	12.64159
chloroplast membrane	0.053379	0.00446	11.9693
chloroplast ribulose bisphosphate carboxylase complex	0.00432	0.000365	11.84351
fatty acid synthase complex	0.00216	0.000182	11.84351
mitochondrial outer membrane	0.025918	0.002188	11.84351
mitochondrial respiratory chain complex III	0.00216	0.000182	11.84351
transcription factor TFIIIB complex	0.00216	0.000182	11.84351
mitochondrial lumen	0.07436	0.006283	11.83458
mitochondrial matrix	0.07436	0.006283	11.83458
chromatin silencing complex	0.001543	0.000133	11.63202
chloroplast envelope	0.081765	0.007062	11.57741
mitochondrial respiratory chain complex I	0.008022	0.000696	11.52124
mitochondrial part	0.253625	0.022182	11.43381
chloroplast thylakoid lumen	0.041962	0.003697	11.35035
mitochondrial envelope	0.195002	0.017308	11.26657
mitochondrial inner membrane	0.149337	0.013346	11.18986
mitochondrial membrane part	0.065103	0.005819	11.18795
glucose-1-phosphate adenylyltransferase complex	0.001851	0.000166	11.16674
mannosyltransferase complex	0.001851	0.000166	11.16674
mitochondrial nucleoid	0.001851	0.000166	11.16674
mitochondrial outer membrane translocase complex	0.003703	0.000332	11.16674
mitochondrial pyruvate dehydrogenase complex	0.003703	0.000332	11.16674
chromatin assembly complex	0.005554	0.000497	11.16674
mitochondrial membrane	0.174637	0.015667	11.14704
photosynthetic membrane	0.220611	0.019795	11.14492
chloroplast thylakoid membrane	0.19963	0.018004	11.08791
chloroplast stroma	0.07004	0.006366	11.00195
chloroplast part	0.35236	0.03203	11.00105
chloroplast thylakoid	0.223696	0.020358	10.9879
mitochondrial respiratory chain	0.029929	0.002735	10.94115
chloroplastic endopeptidase Clp complex	0.00432	0.000398	10.85655

chromatin accessibility complex	0.00216	0.000199	10.85655
mitochondrion	0.825363	0.078516	10.51204
chloroplast	0.893551	0.086258	10.35905
mitochondrial proton-transporting ATP synthase complex	0.007714	0.000746	10.33957
NADH dehydrogenase complex	0.013885	0.001359	10.21348
cell wall	0.297748	0.029228	10.18709
chloroplast stromal thylakoid	0.006479	0.000647	10.02143
mitochondrial respiratory chain complex II	0.002468	0.000249	9.92599
transcription factor TFIIF complex	0.002468	0.000249	9.92599
chloroplast outer membrane	0.011416	0.00116	9.837365
transcription factor complex	0.05955	0.006167	9.655827
transcription factor TFIID complex	0.010491	0.001094	9.587604
mitochondrial small ribosomal subunit	0.003703	0.000398	9.305616
mitochondrial tricarboxylic acid cycle enzyme complex	0.00216	0.000232	9.305616
chromatin remodeling complex	0.034866	0.003863	9.026048
mRNA cleavage factor complex	0.00432	0.000497	8.685241
transcription factor TFIIE complex	0.00216	0.000249	8.685241
mRNA cleavage and polyadenylation specificity factor complex	0.002777	0.000332	8.375054
transcription elongation factor complex	0.005862	0.000713	8.223567
transcription factor TFIIA complex	0.001851	0.000232	7.976242
mitochondrial proton-transporting ATP synthase complex, catalytic core	0.002777	0.000348	7.976242
mitochondrial alpha-ketoglutarate dehydrogenase complex	0.001543	0.000199	7.75468
